# A Lipid with Lewis Pair‐Mediated Targeting and Multiple Stimuli‐Responsive Delivery of Antibiotics for Bacterial Infections

**DOI:** 10.1002/advs.202507407

**Published:** 2025-08-24

**Authors:** Xiaojian Yan, Lei Hua, Hongping Wan, Yong Liu, Tao Jin, Yicen Ge, Michael R Hamblin, Mahdi Karimi, Xinghong Zhao, Yan Hu, Linqi Shi, Yuanfeng Li

**Affiliations:** ^1^ Zhejiang Key Laboratory of Intelligent Cancer Biomarker Discovery and Translation Department of Gynecology Consortium for Infection and Innovation (CII) First Affiliated Hospital Wenzhou Medical University Wenzhou 325035 China; ^2^ Translational Medicine Laboratory The First Affiliated Hospital of Wenzhou Medical University Wenzhou Medical University Wenzhou Zhejiang 325001 China; ^3^ State Key Laboratory of Medicinal Chemical Biology Nankai University Tianjin 300071 China; ^4^ Center for Sustainable Antimicrobials Center for Infectious Diseases Control (CIDC) Department of Pharmacy College of Veterinary Medicine Sichuan Agricultural University Chengdu 611130 China; ^5^ Wenzhou Institute University of the Chinese Academy of Sciences Wenzhou Zhejiang 325001 China; ^6^ College of Materials Chemistry and Chemical Engineering Chengdu University of Technology Chengdu Sichuan 610059 China; ^7^ Laser Research Centre University of Johannesburg Doornfontein Johannesburg 2028 South Africa; ^8^ Cellular and Molecular Research Center Iran University of Medical Sciences Tehran 14535 Iran

**Keywords:** bacterial biofilms, drug delivery, dynamic covalent bonding, lipid nanoparticles, multiple responsiveness

## Abstract

Integrating both targeted delivery and stimulus‐responsive release into a single small molecule for drug delivery presents challenges related to synthesis, stability, and efficacy. In this study, single‐molecular lipids incorporating a Lewis pair (*Lp*‐lipids) are described, composed of a Lewis acid (phenylboronic acid) and a Lewis base (amine) within a single small molecular structure, to formulate lipid nanoparticles for antibiotic delivery. For targeted delivery to bacterial biofilms, the phenylboronic acid selectively binds to bacteria or biofilms by forming boronate ester bonds with diols in the microbial dextran or peptidoglycan. Additionally, the amine group responds to the acidic microenvironment, enhancing electrostatic interactions with bacteria and biofilms. Regarding stimulus‐responsive drug release, the Lewis base reacts to low pH, while the Lewis acid responds to H_2_O_2_ and ATP, triggering changes in the hydrophobicity and structural integrity of the lipid nanoparticles. These changes facilitate the release of encapsulated antibiotics, effectively eradicating both Gram‐positive and Gram‐negative bacteria in vitro and in vivo. The combined targeting and stimuli‐responsive release properties of *Lp*‐lipids significantly enhance their potential for biomedical applications and clinical translation.

## Graphical abstract

Lewis pair‐integrated lipids (*Lp*‐lipids), combining targeted delivery and stimuli‐responsive are developed for antibiotic delivery. These lipids form nanoparticles that bind biofilms via boronate ester bonds and respond to acidic pH, H_2_O_2_, and ATP, enabling controlled drug release. *Lp*‐lipids effectively eradicate bacteria, showing promise for clinical translation.

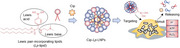

## 1 Introduction

Nanocarriers are gaining widespread attention and increased application in drug delivery, offering advanced solutions for precise therapeutic targeting and controlled drug release.^[^ ^1^, ^2^ ^]^ The primary objective of targeted and responsive drug release in nanocarriers is to enhance therapeutic efficacy while minimizing potential side effects.^[^ ^3^ ^]^ By specifically directing the drug to target cells or tissues, nanocarriers ensure that the therapeutic agent is delivered exactly where it is needed, thus enhancing its potency against the intended disease while minimizing exposure to healthy tissues and reducing toxic side effects.^[^ ^4^ ^]^ Responsive drug release is equally important, enabling the drug to be released in a controlled manner upon encountering specific environmental triggers, such as pH changes, temperature fluctuations, or the presence of certain biomolecules.^[^ ^5^, ^6^, ^7^, ^8^ ^]^ This stimulus‐responsive property can trigger drug release in the physiological conditions associated with the disease state, allowing for a more on‐demand therapeutic approach.

In designing nanocarriers, it is common to incorporate distinct domains for targeting and stimulus‐responsiveness, each contributing to the overall function of the delivery system. For instance, in lipid nanoparticle systems, one lipid type may serve as the targeting agent, while one or two additional lipids provide stimuli‐responsive properties.^[^ ^9^, ^10^ ^]^ However, the combination of targeting and responsive release functionalities within a single molecular structure poses significant challenges. Effective targeting often requires surface modifications or the incorporation of ligands capable of recognizing and binding to specific cellular receptors, while responsive release mechanisms rely on materials that react selectively to environmental stimuli.^[^ ^11^, ^12^ ^]^ Balancing these two functionalities within a single system can complicate the synthesis, affect stability, and introduce competing interactions that may reduce the efficacy of targeting or release.

Recently, in some polymer‐based or peptide‐based systems,^[^ ^13^, ^14^, ^15^, ^16^ ^]^ researchers have attempted to combine targeting and stimulus‐responsiveness within a single small molecule. While these multifunctional systems show potential, they often require labor‐intensive synthetic procedures and face challenges related to scalability and cost‐effectiveness, limiting their clinical applicability.^[^ ^17^ ^]^ Consequently, the development of small molecules that can seamlessly integrate both targeted delivery and stimuli‐responsive release is a complex yet essential goal in advancing nanoparticle‐based therapies.^[^ ^18^ ^]^ Such innovations hold promise for creating more efficient, versatile, and clinically viable drug delivery systems, opening new avenues for precision medicine to treat complex diseases.

**Scheme 1:**
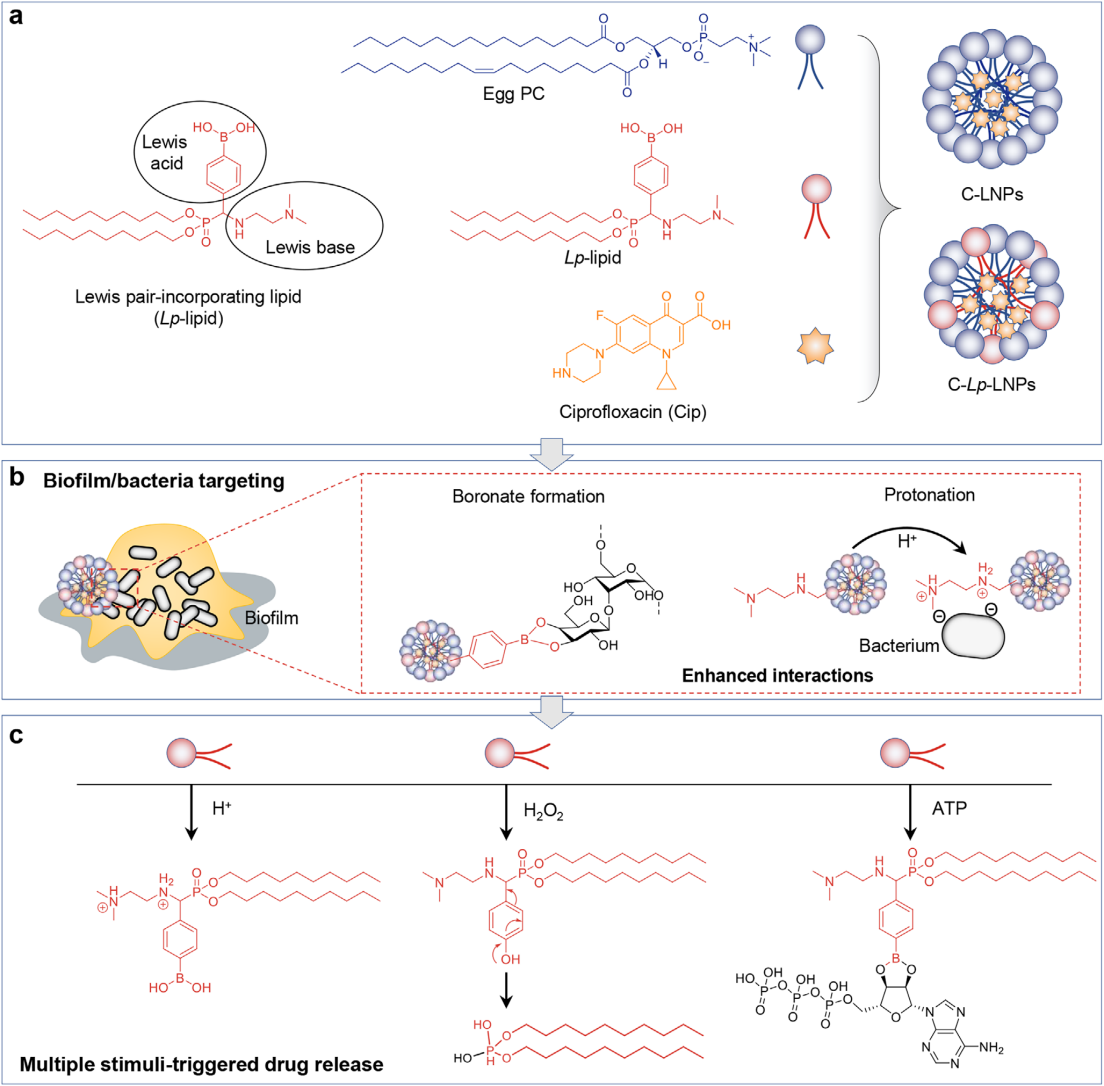
Schematic illustration depicting the formation of C‐LNPs and C‐*Lp*‐LNPs, along with the biofilm/bacteria binding capacity of C‐*Lp*‐LNPs and the triple stimuli‐responsive drug release in the presence of acid, H_2_O_2_, and ATP. a) Chemical structures of Egg PC, *Lp*‐lipid, and Cip utilized in the fabrication of C‐LNPs and C‐*Lp*‐LNPs. The distinction between C‐LNPs and C‐*Lp*‐LNPs lies in the incorporation of Cip in C‐*Lp*‐LNPs. The weight ratio of *Lp*‐lipid to Egg PC was set at 10/90. b) C‐*Lp*‐LNPs exhibit binding to bacteria and their biofilms through the dynamic interactions between *Lp*‐LNPs and the diols on dextran and peptidoglycans, as well as electrostatic interactions between *Lp*‐LNPs with bacteria. c) *Lp*‐lipid's response to acidity, H_2_O_2_, and ATP within the bacteria‐infected microenvironment triggers a change in hydrophobicity of *Lp*‐lipid, ultimately leading to drug release from C‐*Lp*‐LNPs.

Herein, we report the synthesis of lipids incorporating a separated Lewis pair (*Lp*‐lipids)—comprising a boronic acid as the Lewis acid and a secondary or tertiary amine as the Lewis base—within a single small molecular framework. This unique structure facilitates the dual functions of targeted delivery and stimulus‐responsive release of antibiotics, enhancing the efficacy of treating bacterial infections (**Scheme** **1**). Specifically, these lipids serve as key components in the formulation of lipid nanoparticles (LNPs) containing egg phosphatidylcholine (Egg PC) to encapsulate ciprofloxacin (Cip) (Scheme 1a). The separated Lewis pair offers several distinct advantages. Firstly, the phenylboronic acid domain provides additional hydrophobic interactions, stabilizing the lipid nanoparticles and improving drug loading efficiency through enhanced drug‐carrier interactions, such as π‐π stacking. In terms of targeting infection sites, phenylboronic acid facilitates selective binding to bacteria or bacterial biofilms by dynamically forming boronate ester bonds with diols present in dextran or peptidoglycan.^[^ ^19^, ^20^ ^]^ Additionally, the acidic microenvironment generated by bacterial metabolism further enhances electrostatic interactions with bacteria and biofilms because it induces a more positively charged surface on the lipid nanoparticles (Scheme 1b). Regarding stimuli‐responsive drug release, the lipids containing separated Lewis pairs are responsive to various stimuli, including low pH, elevated H_2_O_2_ levels, and adenosine triphosphate (ATP) (Scheme 1c).^[^ ^21^, ^22^, ^23^ ^]^ The Lewis base responds to acidity, while the Lewis acid domain reacts with H_2_O_2_ and ATP, leading to changes in the hydrophobicity and integrity of the LNPs. These changes ultimately trigger the release of encapsulated antibiotics, enabling precise, targeted, and controlled eradication of embedded bacteria. This approach offers a promising strategy for combating bacterial infections.

## 2 Results and Discussion

### 2.1 Preparation of Multiple Responsiveness of *Lp*‐LNPs

Initially, the *Lp*‐lipids were synthesized following the synthetic route outlined in Figure S1, Supporting Information, using a two‐step, one‐pot reaction with a minor modification from our previous publications.^[^ ^24^, ^25^ ^]^ Lipids with varying carbon chain lengths (10C and 14C) were prepared, and their chemical structures were confirmed through their corresponding NMR spectra (Figures S2–S5, Supporting Information) prior to further studies.

**Figure 1:**
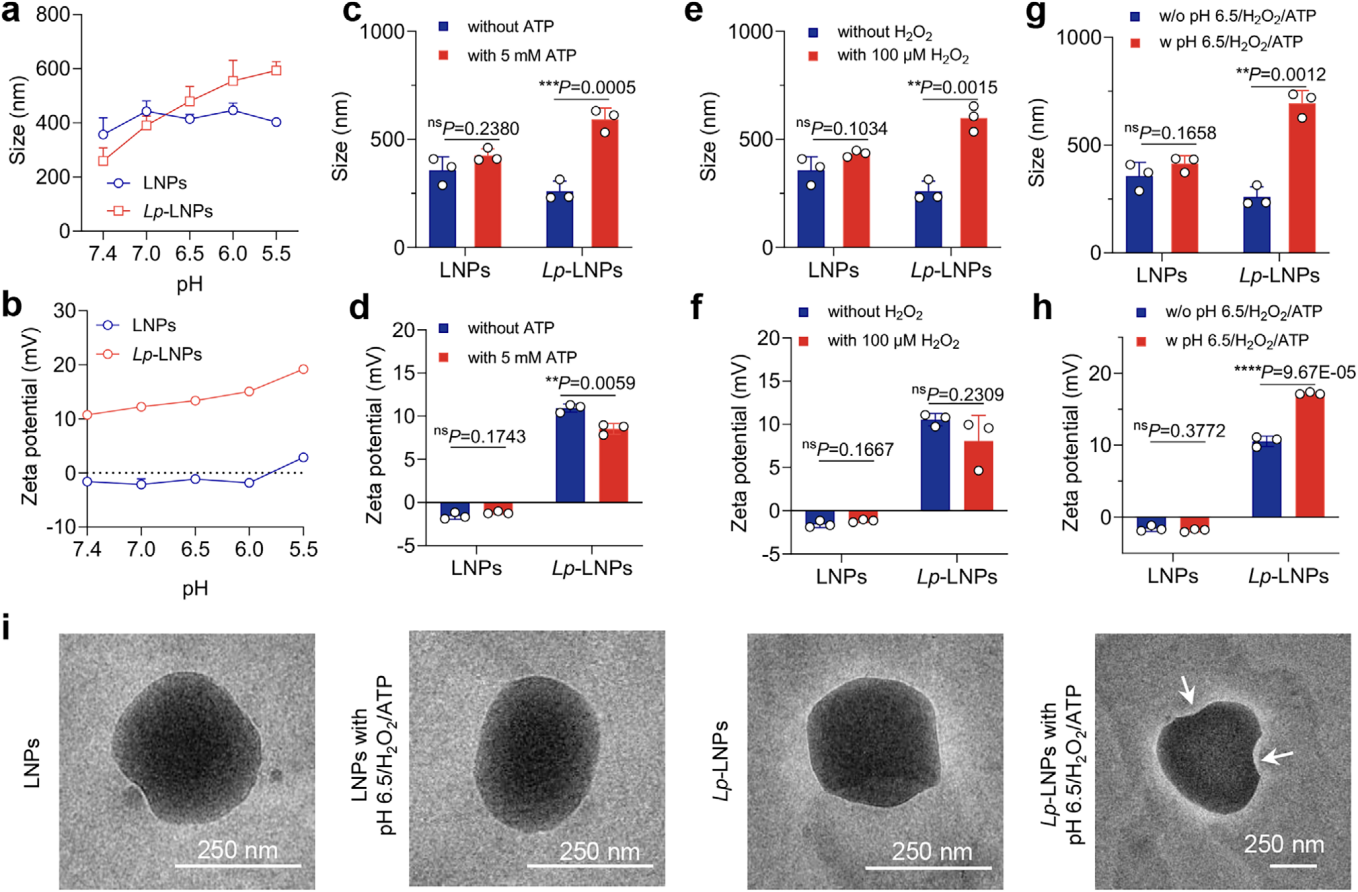
a) Size variations of LNPs and *Lp*‐LNPs upon exposure to pH levels ranging from 7.4 to 5.5. Data are presented as mean ± standard deviations over three replicates. Size changes of LNPs and *Lp*‐LNPs in the absence or presence of c) 5 mM ATP, e) 100 µM H_2_O_2_, and g) pH 6.5/ H_2_O_2_/ATP for 2 h, emulating the acidic/oxidative/high ATP microenvironment present in bacterial infections. b) Zeta potentials of LNPs and *Lp*‐LNPs upon exposure to pH levels ranging from 7.4 to 5.5. Data are presented as mean ± standard deviations over three replicates. Size changes of LNPs and *Lp*‐LNPs in the absence or presence of d) 5 mM ATP, f) 100 µM H_2_O_2_, and h) pH 6.5/ H_2_O_2_/ATP for 2 h, emulating the acidic/oxidative/high ATP microenvironment present in bacterial infections. Size and zeta potentials were measured using the dynamic light scattering method, with the nanoparticle concentration set at 100 µg mL^−1^ during the measurements. i) Representative TEM images of the LNPs and *Lp*‐LNPs before and after exposure to pH 6.5/ H_2_O_2_/ATP for 2 h, with white arrows indicating the defects of *Lp*‐LNPs observed in the nanoparticles following the stimulation.

To prepare the *Lp*‐LNPs, we utilized a weight ratio of 90:10 between Egg PC and *Lp*‐lipid, a ratio commonly reported in prior publications,^[^ ^26^ ^]^ employing a thin film hydration followed by an extrusion method. Egg PC is a phospholipid derived from egg yolk, frequently used to create drug delivery vehicles that enhance drug stability, prolong circulation time, and allow targeted delivery to specific tissues, thereby improving drug efficacy and minimizing side effects.^[^ ^27^ ^]^ For control purposes, LNPs devoid of *Lp*‐lipid were prepared. The as‐prepared LNPs had a diameter of 357.0 ± 50.8 nm, whereas the incorporation of *Lp*‐lipid resulted in a reduced diameter of 259.7 ± 39.0 nm (**Figure** **1**a), likely due to the compacting effect of *Lp*‐lipids on the LNPs.^[^ ^28^ ^]^ The zeta potential of the freshly prepared LNPs was –1.6 ± 0.3 mV, indicating a neutral surface charge. Notably, the *Lp*‐LNPs displayed a positive charge, with a zeta potential of 10.7 ± 0.4 mV (Figure 1b). This positive surface charge can be attributed to the presence of protonated tertiary amines in *Lp*‐LNPs at pH 7.4, in accordance with the estimated pKa values (Figure S6, Supporting Information).^[^ ^29^ ^]^

In bacterial infections, the acidic microenvironment is primarily caused by bacterial metabolic byproducts, such as lactic acid.^[^ ^30^ ^]^ Furthermore, the immune response to the infection (particularly neutrophils) contributes to increased acidity as a defense mechanism to inhibit bacterial growth.^[^ ^31^ ^]^ The elevated concentration of H_2_O_2_ in the infected site, reaching up to 1 mM, comes from the oxidative burst generated by neutrophils and other immune cells to combat the invading pathogens,^[^ ^32^, ^33^ ^]^ serving as a key component of the host's defense strategy. Additionally, extracellular ATP, a host‐derived signaling molecule, is released in concentrations of 5–10 mM by stressed or injured epithelial cells at the infection site,^[^ ^21^, ^34^ ^]^ as a signal of the presence of invading pathogens and alerting the host immune system.

Subsequently, we investigated the reaction of *Lp*‐lipids to various stimuli within the bacterial infection microenvironment. After gradually adjusting the surrounding pH to an acidic level (pH 6.5), the size and zeta potentials of LNPs remained relatively unchanged. However, under the same conditions, the size of *Lp*‐LNPs increased to ≈600 nm, while the zeta potential rose to over 20 mV (Figure 1c,d), indicating the protonation of additional amines in the *Lp*‐lipids and a shift in the hydrophobicity of the *Lp*‐lipids. Following treatment with ATP (5 mM), the size and zeta potential of LNPs exhibited only marginal changes. In contrast, *Lp*‐LNPs experienced a marked increase in size to ≈700 nm, and their surface charge decreased to 7 mV, attributed to the binding of ATP to the *Lp*‐lipid. Furthermore, in the presence of 100 µM H_2_O_2_,^[^ ^28^ ^]^ the size and zeta potential of *Lp*‐LNPs changed dramatically, while LNPs showed no significant alterations in size or zeta potential. When all three stimuli were combined, *Lp*‐LNPs exhibited a two‐fold increase in size and a 50% rise in zeta potential, outperforming LNPs under identical conditions (Figure 1e,f). Upon treatment with pH 6.5/H_2_O_2_/ATP, *Lp*‐LNPs showed pronounced changes in size and zeta potentials compared to those receiving single stimuli (Figure 1g,h). When utilizing *Lp*‐lipid with a carbon chain length of 14, similar results were observed (Figure S7, Supporting Information). However, it is noteworthy that the changes in size and zeta potential of the *Lp*‐lipid with a carbon chain length of 14 did not exhibit the same extent of variation as those of the *Lp*‐lipid with a carbon chain length of 10 upon treatment with pH 6.5/H_2_O_2_/ATP. This discrepancy may be attributed to the longer carbon chain length, which may enhance the stability of the LNPs and mitigate changes in size and morphology. These findings align with our previous studies, which demonstrated that lipids with a 10C chain length outperformed those with other chain lengths.^[^ ^25^ ^]^ Accordingly, in our subsequent investigations, we opted to employ the *Lp*‐lipid with a 10‐carbon chain for most of our further studies.

Transmission electron microscopy (TEM) images further confirmed the solid structures of both LNPs and *Lp*‐LNPs. Upon treatment with pH 6.5/H_2_O_2_/ATP, LNPs did not exhibit significant changes in nanoparticle morphology; however, the treatment with pH 6.5/H_2_O_2_/ATP induced both an increase in size and the appearance of defects in the *Lp*‐LNPs structures(Figure 1i; Figure S8, Supporting Information).

Additionally, we examined the responsiveness of *Lp*‐LNPs to glucose. After exposure to glucose at a concentration of 0.1 mg mL^−1^ (normal blood glucose level), no significant change in size was observed. However, when exposed to glucose at a concentration of 0.2 mg mL^−1^ (diabetic blood glucose level), the size of *Lp*‐LNPs increased from 200 to 600 nm (Figure S9, Supporting Information). This suggests that normal blood glucose levels would not interfere with the effectiveness of *Lp*‐LNPs, while elevated glucose levels, such as those found in diabetes, may impact their performance. Colloidal stability showing that *Lp*‐LNPs remain stable at physiological pH for at least 5 days (Figure S10, Supporting Information). Future studies could further explore this interaction and assess strategies to optimize *Lp*‐LNP performance under varying physiological conditions, particularly in patients with diabetes.

### 2.2 Mechanism Studies

**Figure 2:**
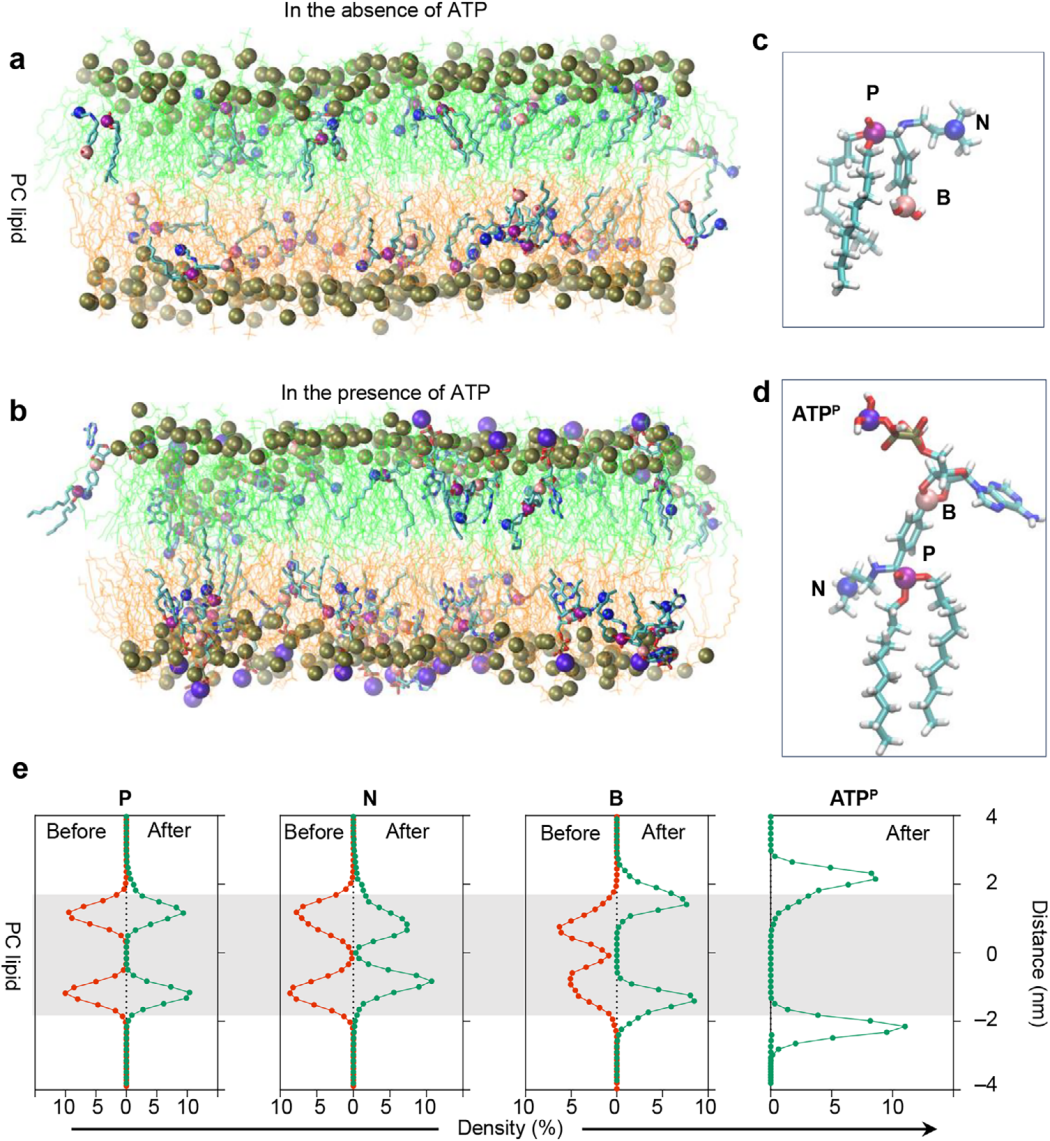
All‐atom simulation to study the change of structure and atomic density distribution in the presence of ATP. Snapshots of the bilayer membrane composed of Egg PC and *Lp*‐lipids in the absence of ATP a) and in the presence of ATP b) after a 100‐ns simulation. c, d) Snapshots illustrating the configurations of *Lp*‐lipids in the absence of ATP (c) and in the presence of ATP (d). Different atoms are represented by beads of distinct colors: phosphorus (**P**) as purple, nitrogen (**N**) as blue, boron (**B**) as pink, and the third phosphorus atom in ATP (**ATP^P^**) as violet. e) Atomic density distribution curves of **P**, **N**, **B**, and **ATP^P^** of *Lp*‐lipids in the bilayer membrane before and after exposure to ATP, demonstrating that the polar heads of ATP‐anchored *Lp*‐lipids are positioned on the surface of the bilayer membrane. The gray background denotes the thickness of the PC bilayer membrane.

To investigate the impact of various stimuli on the stability and physicochemical properties of *Lp*‐LNPs, an all‐atom simulation was performed (**Figure** **2**). For simplification, a lipid bilayer model was utilized instead of a real sphere. By following the evolution of membrane thickness throughout the simulation, we observed that the introduction of *Lp*‐lipid resulted in a thinner PC lipid membrane, corroborating our earlier findings that the presence of *Lp*‐lipid renders the nanoparticles more compact (Figure S11, Supporting Information). By analyzing the interaction energies between Egg PC/Egg PC and Egg PC/*Lp*‐lipid molecules during the 100 ns co‐assembly process, we found that the interaction energy between Egg PC and *Lp*‐lipid molecules was significantly lower than that between Egg PC molecules alone, indicating stronger interactions in the Egg PC/*Lp*‐lipid pair (Figure S12, Supporting Information). The compact structure and smaller initial size of *Lp*‐LNPs can be attributed to hydrophobic interactions among lipid chains, as well as possible π–π stacking interactions between aromatic groups—such as lipophilic prodrug moieties or phenylboronic acid—within the lipid bilayer. These interactions facilitate tight molecular packing, leading to the formation of smaller and more compact nanoparticles. Further atomic‐level analysis of the density distribution within the bilayer membrane, upon incorporation of *Lp*‐lipid and in the presence of ATP, provided additional structural insights (Figure 2a–d).

The phosphorus (P) atoms in the *Lp*‐lipids were situated deeper within the membrane compared to those in Egg PC, contributing to the observed variation in membrane thickness (Figure 2e). The positioning of nitrogen (N) and boron (B) atoms in the *Lp*‐lipids showed a different arrangement when ATP was present. Specifically, the N atom in the *Lp*‐lipid, being more hydrophilic, was positioned outward relative to the B atom. However, the addition of ATP altered the distribution of hydrophilic and hydrophobic characteristics within the molecule. The increased hydrophilicity of the ATP molecule drew the B atom closer to the membrane surface, resulting in a corresponding inward shift of the N atom's position (Figure 2c,d). Furthermore, the P atom of ATP (ATP^P^) was distributed more outwardly, as visually demonstrated by the protruding purple beads on the membrane surface in the accompanying configuration snapshot.

**Figure 3:**
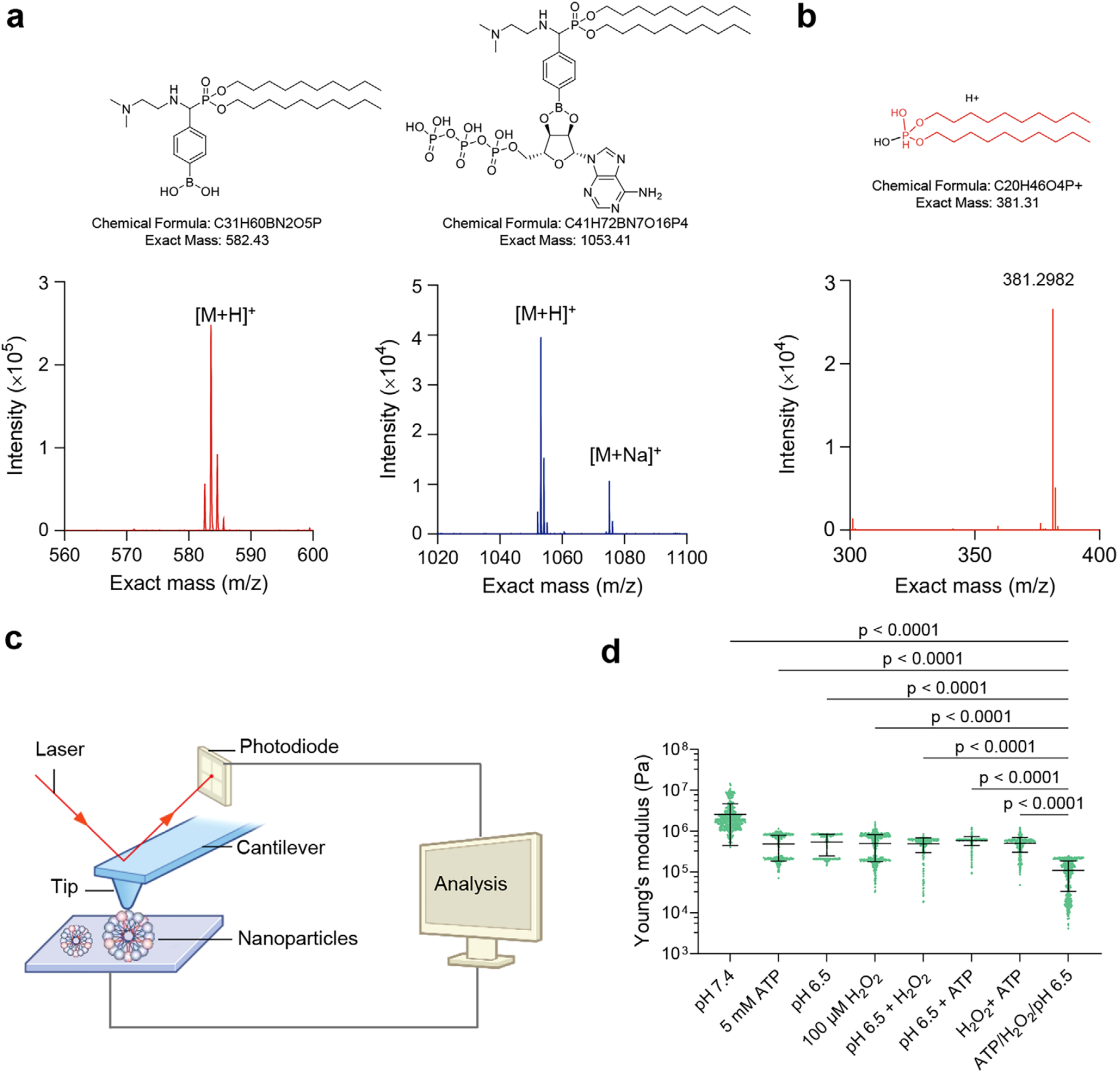
Stimulus‐responsive behavior of *Lp*‐lipids. a) Matrix‐assisted laser desorption/ionization time‐of‐flight (MALDI‐TOF) mass spectrometry of *Lp*‐lipids before and after exposure to ATP (5 mM) for 2 h, illustrating the formation of a boronate structure between *Lp* in *Lp*‐lipids and the cis‐diol in ATP. b) Electrospray ionization (ESI) mass spectrometry reveals the products of *Lp*‐lipid after exposure to H_2_O_2_ (100 µM) for 2 h. c) Schematic illustration of the bio‐atomic force microscopy technique employed to measure the mechanical properties of *Lp*‐LNPs following exposure to various stimuli. d) Young's modulus of *Lp*‐LNPs after exposure to various treatments, determined via the bioAFM technique.

In summary, our simulation provides a comprehensive overview of how the introduction of *Lp*‐lipids, both in the absence and presence of ATP, influences the structural evolution and atomic density distribution of the bilayer membrane. These visualizations illuminate the intricate interplay between membrane components and offer a deeper understanding of the system dynamics under investigation. As a proof of concept, the formation of the *Lp*‐lipid and ATP conjugate was validated by the presence of a distinct mass peak in the Matrix‐Assisted Laser Desorption/Ionization Time‐of‐Flight (MALDI‐TOF) measurement (**Figure** **3**a). Similar results were observed with *Lp*‐lipid featuring a 14C chain length, further confirming the successful conjugation between *Lp*‐lipid and ATP (Figure S13, Supporting Information). These findings provide compelling evidence in favor of the formation of the *Lp*‐lipid and ATP conjugate.^[^ ^35^ ^]^

Moreover, by mass spectrometry analysis, we verified that *Lp*‐lipid could generate phosphoric acid upon exposure to H_2_O_2_ (as shown in Figure 3b). The potential reaction mechanism is detailed in Figure S14, Supporting Information. Subsequently, we employed bio‐atomic force microscopy (bioAFM) to assess changes in the mechanical properties of *Lp*‐LNPs under various treatment conditions (Figure 3c). In phosphate buffer alone, *Lp*‐LNPs exhibited a broad Young's modulus distribution. Exposure to single stimuli resulted in more concentrated distributions, indicating more uniform mechanical properties. None of the dual‐stimuli combinations induced a significantly enhanced response compared to single stimuli; although slight increases were observed, they were not statistically significant. In contrast, the triple‐stimuli condition led to a much broader distribution, suggesting increased mechanical heterogeneity. Quantitative analysis revealed a decrease in Young's modulus from 10^6^ to 10^4^ Pa under triple‐stimuli conditions, indicating greater deformability (Figure 3d). These findings demonstrate the ability of *Lp*‐LNPs to dynamically adjust their mechanical properties in response to complex environmental cues, enhancing their potential for targeted therapeutic applications.

### 2.3 Interactions of *Lp*‐LNPs with Bacteria

**Figure 4:**
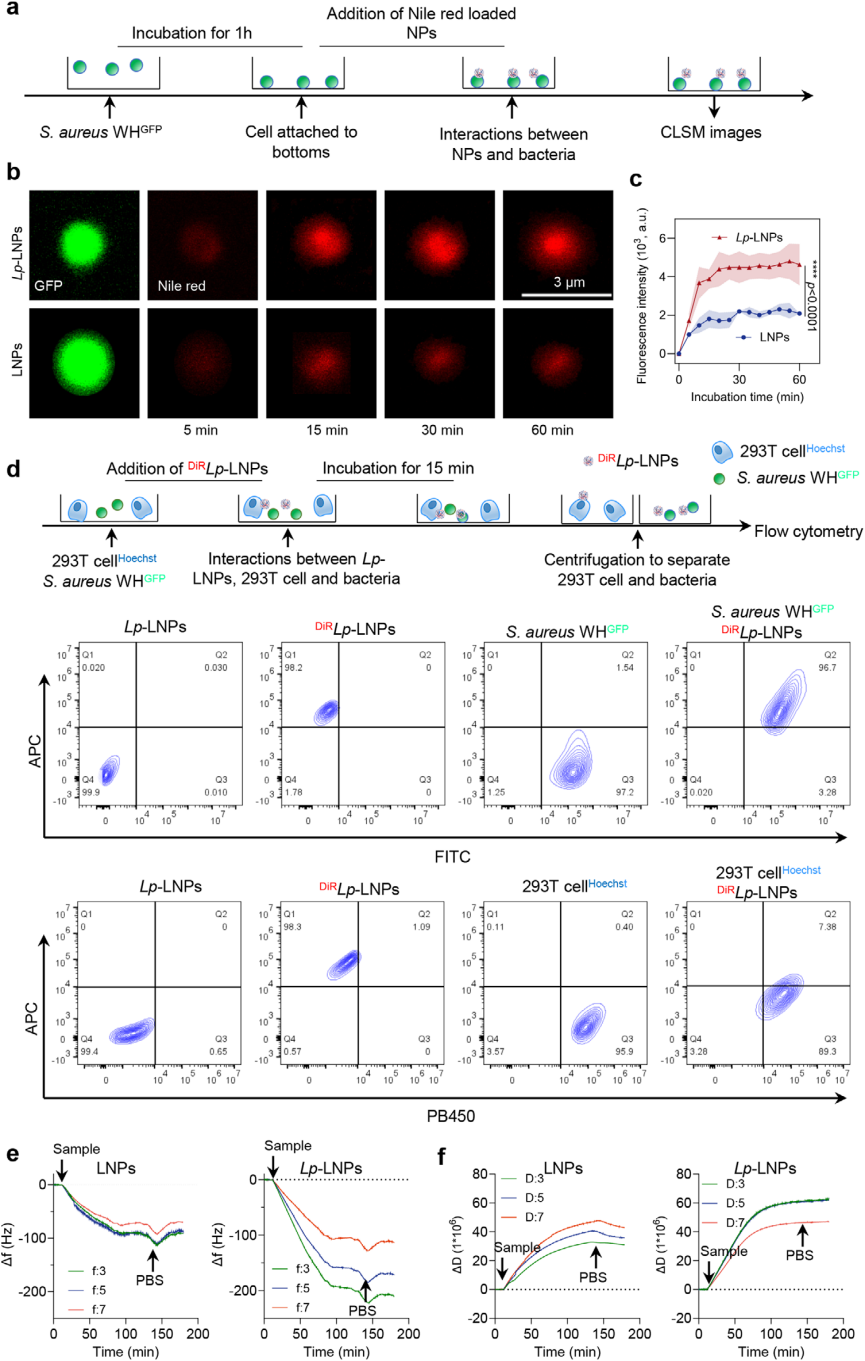
*Lp*‐LNPs show fast and strong binding to bacteria. a) Workflow illustrating the experimental process for investigating the interactions of Nile red‐loaded *Lp*‐LNPs with *S. aureus* WH^GFP^ using CLSM. Note that bacteria and nanoparticles are not depicted to scale. b) Representative images from the green channel (GFP‐expressing bacteria) and red channel (Nile red‐loaded LNPs) of *S. aureus* WH^GFP^ after interaction with Nile red‐loaded *Lp*‐LNPs over a 1‐h incubation period. c) Quantified fluorescence intensity of the red channel from a single bacterium after exposure to Nile red‐loaded LNPs and *Lp*‐LNPs. Data are presented as mean ± standard deviations across three replicates. d) Workflow and subsequent flow cytometry profiles demonstrating the selective interactions of DiR‐loaded *Lp*‐LNPs with *S. aureus* WH^GFP^ versus 293T cells. e) QCM‐D analysis of the frequency shift (Δf) observed for the third, fifth, and seventh overtones resulting from the adsorption of LNPs and *Lp*‐LNPs on *S. aureus*‐coated sensors at a flow rate of 0.15 mL min^−1^. f) QCM‐D analysis of the dissipation (ΔD) observed for the third, fifth, and seventh overtones from the adsorption of LNPs and *Lp*‐LNPs on *S. aureus*‐coated sensors at a flow rate of 0.15 mL min^−1^. NP indicates the introduction of LNPs or LNPs into the flow chamber, while PBS signifies a washing step. For further details regarding the preparation of each substrate, please refer to the experimental section. Representative curves are shown from experiments conducted in triplicate.

We further investigated the interactions between LNPs/*Lp*‐LNPs and bacteria, beginning with confocal laser scanning microscopy (CLSM) and using *Staphylococcal aureus* WH^GFP^ as a model strain (**Figure** **4**a). Our observations revealed that *Lp*‐LNPs rapidly congregated around the bacteria, in stark contrast to the relatively slow accumulation of LNPs (Figure 4b). Upon quantification, it became evident that *Lp*‐LNPs bound to the bacteria within just 10 min (Figure 4c), demonstrating markedly superior bacterial targeting compared to their LNP counterparts. Additionally, flow cytometry analysis conducted after a 2‐h co‐incubation with bacteria confirmed these findings, revealing that 76.2% of the *Lp*‐LNPs were colocalized with *S. aureus* WH^GFP^ in quadrant Q2 (Figure S15, Supporting Information), suggesting the binding of *Lp*‐LNPs to bacteria. On the contrary, LNPs, in the absence of a Lewis pair, demonstrated marginal interaction with bacteria (Figure S16, Supporting Information). To investigate the selectivity of our *Lp*‐LNPs against bacteria and mammalian cells, we co‐cultured red fluorescent‐labeled *Lp*‐LNPs with green fluorescent *S. aureus* WH^GFP^ and blue fluorescent 293T cells. After separation, we observed that the majority of *Lp*‐LNPs were associated with *S. aureu*s WH^GFP^ rather than 293T cells (Figure 4d). This suggests that our *Lp*‐LNPs selectively target *S. aureus* WH^GFP^, likely due to the denser polysaccharides and negatively charged surface of the bacterial cells.

We subsequently employed quartz crystal microbalance with dissipation (QCM‐D) monitoring, a highly sensitive technique to investigate the mass and viscoelastic properties of thin films or layers on the surface of a quartz crystal.^[^ ^36^ ^]^ We used QCM‐D to examine the interactions between bacteria and *Lp*‐LNPs (Figure 4e,f). The change in frequency (Δf) is directly proportional to the change in mass (Δm) of the adsorbed layer.^[^ ^37^ ^]^ Our findings revealed that *Lp*‐LNPs induced a more pronounced frequency shift on bacteria‐attached sensors compared to LNPs, suggesting greater adsorption of *Lp*‐LNPs to these surfaces. Furthermore, a higher dissipation factor (D) was observed on the *Lp*‐LNPs‐treated bacteria‐attached sensors, indicating the presence of a more viscous or less rigid layer. This suggests that the layers formed through *Lp*‐LNP absorption could be thicker, softer, or more hydrated. The combined analysis of frequency shifts and dissipation changes provides a comprehensive understanding of the characteristics of *Lp*‐LNP absorption on bacteria‐attached sensors, encompassing their thickness, density, and mechanical properties. The significant colocalization and high affinity proved by the above experiments underscore the enhanced targeting capability of *Lp*‐LNPs, highlighting their potential for effective applications in bacterial targeting and therapeutic delivery.

### 2.4 Interactions of *Lp*‐LNPs with Bacterial Biofilms

**Figure 5:**
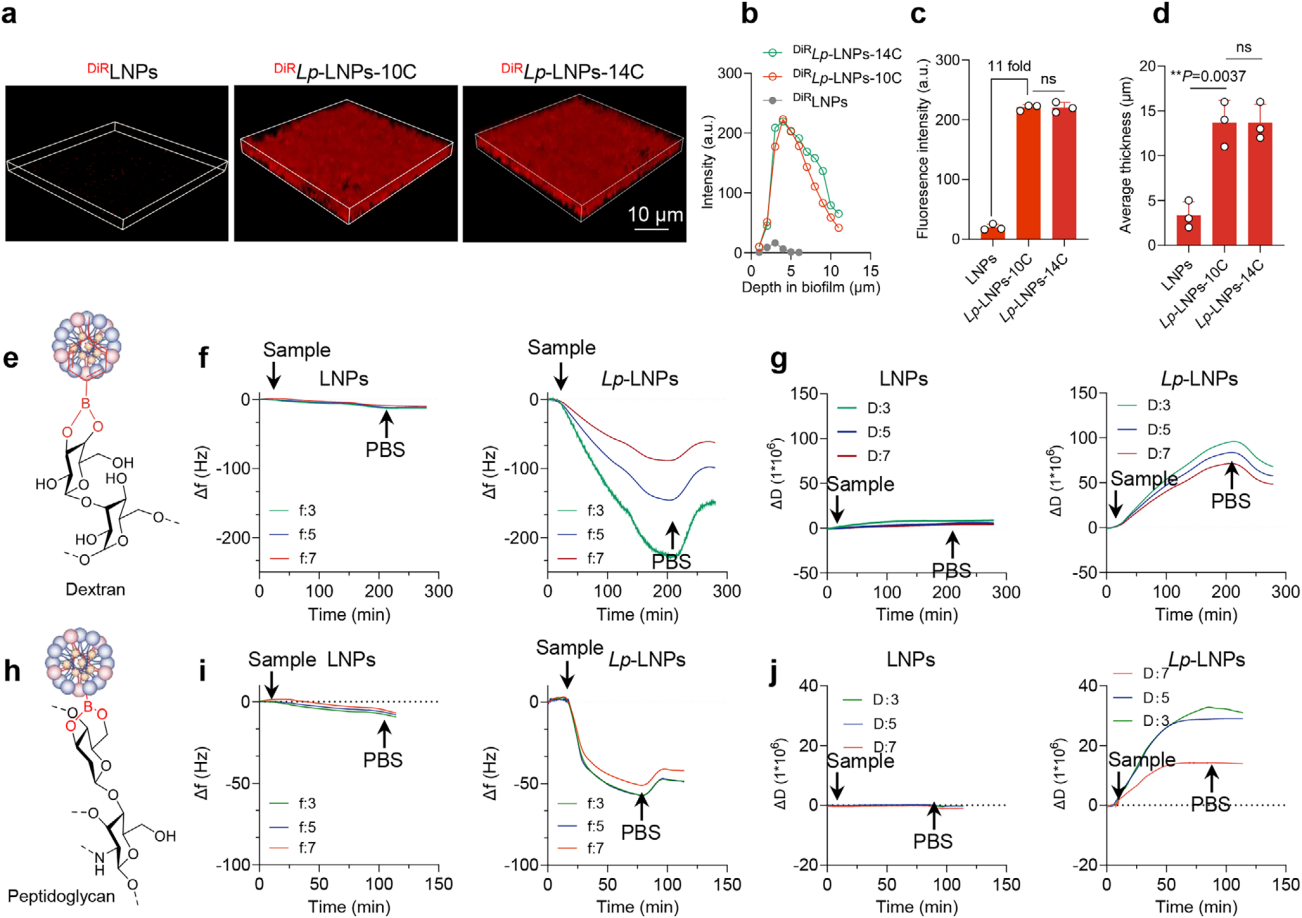
*Lp*‐LNPs exhibit rapid and robust interactions with *S. aureus* biofilms. a) Representative images from the CLSM red channel of *S. aureus* biofilms after a 2‐h exposure to DiR‐loaded LNPs and *Lp*‐LNPs with 10C or 14C chain length. For clarity, only the red channels indicating the presence of nanoparticles are displayed. b) Quantified fluorescence intensity from the CLSM planes shown in (a) as a function of biofilm depth after exposure to DiR‐loaded LNPs and *Lp*‐LNPs with 10C or 14C chain length. c) Total fluorescence intensity within the *S. aureus* biofilms following a 2‐h exposure to DiR‐loaded LNPs and *Lp*‐LNPs with 10C or 14C chain length. d) Average thickness demonstrating the penetration of DiR‐loaded LNPs and *Lp*‐LNPs with 10C or 14C chain length into *S. aureus* biofilms. e) Illustration of the interactions between *Lp*‐LNPs and dextran via boronate bonding. f, g) QCM‐D analysis of Δf (f) and ΔD (g) observed for the third, fifth, and seventh overtones resulting from the adsorption of LNPs and *Lp*‐LNPs on dextran‐coated sensors at a flow rate of 0.15 mL min^−1^. h) Illustration of the interactions between *Lp*‐LNPs and peptidoglycan via boronate bonding. i, j) QCM‐D analysis of Δf (i) and ΔD (j) observed for the third, fifth, and seventh overtones from the adsorption of LNPs and *Lp*‐LNPs on peptidoglycan‐coated sensors at a flow rate of 0.15 mL min^−1^.

Bacterial biofilms are intricate, structured communities of microorganisms that adhere to a surface. The cells are embedded within a protective extracellular matrix composed of polysaccharides, proteins, and nucleic acids.^[^ ^38^, ^39^ ^]^ This matrix not only shields the bacteria from environmental threats and antimicrobial compounds but also facilitates intercellular communication and nutrient exchange.^[^ ^20^, ^40^ ^]^ The incorporation of boronic acid domains presents a strategic approach to target bacterial biofilms,^[^ ^41^, ^42^ ^]^ as these domains can specifically bind to the diol groups present in the polysaccharide in the extracellular polymeric substances of biofilm, thereby enhancing affinity for biofilm structures. To verify this, we first mixed DiR‐loaded LNPs with 2‐day‐old *S. aureus* Xen36 biofilms. Analysis of the corresponding CLSM images revealed a significantly higher amount of nanoparticles in biofilms treated with *Lp*‐LNPs (**Figure** **5**a,b). Upon quantification, we observed that 11‐fold more *Lp*‐LNPs were bound to the bacterial biofilms, compared to LNPs (Figure 5c). Additionally, we discovered that *Lp*‐LNPs penetrated 4‐fold deeper into the biofilms than LNPs (Figure 5d). To explore the structure–function relationship, we compared C10‐ and C14‐based *Lp*‐LNPs and found no significant difference in their biofilm penetration or antimicrobial activity under the tested conditions (Figure 5a–d). Both formulations exhibited similar diffusion profiles in our in vitro model. The decrease in fluorescence intensity observed at greater depths (Figure 5b) is likely due to increased biofilm density and optical attenuation within the thick biofilm matrix. To isolate the contribution of the Lewis pair design, we constructed positively charged liposomes using 1,2‐dioleoyl‐3‐trimethylammonium‐propane (DOTAP) without the phenylboronic acid domain (LNP‐DOTAP). Compared to LNP‐DOTAP, *Lp*‐LNPs exhibited approximately threefold higher biofilm penetration and retention, indicating that the enhanced biofilm interaction is not solely due to surface charge. These results highlight the critical role of the phenylboronic acid domain in promoting deep biofilm access (Figure S17, Supporting Information).

To further investigate the binding kinetics of *Lp*‐LNPs to bacterial biofilms, we employed QCM‐D measurements. For clarity and simplification, we utilized two key polysaccharides present in biofilms, namely dextran and peptidoglycan, to coat the sensors. Our experiments indicated that LNPs, in the absence of *Lp*, exhibited negligible binding to the dextran‐ or peptidoglycan‐coated surfaces (Figure 5e–g). In contrast, *Lp*‐LNPs induced a drastic shift in frequency on both dextran and peptidoglycan‐coated sensors, measuring two orders of magnitude higher than that of LNPs. Furthermore, *Lp*‐LNPs bound rapidly to the dextran‐ or peptidoglycan‐coated surfaces within 30 min, indicating the rapid formation of *Lp*/ci‐diol complexes (Figure 5h–j). Subsequent dissipation factor measurements suggested that *Lp*‐LNPs create a more viscous or less rigid layer on the dextran‐ and peptidoglycan‐coated surfaces. Additional experiments using Glypican‐3 (GPC3), a representative component of the mammalian glycocalyx that is rich in glycosaminoglycans, showed that the interaction between *Lp*‐LNPs and GPC3 was significantly weaker compared to the interaction with bacterial extran and peptidoglycan (Figure S18, Supporting Information). Collectively, this suggests that our LNPs exhibit a higher binding affinity toward bacterial biofilms than toward mammalian glycan structures. Our results indicate that *Lp*‐LNPs can efficiently target and penetrate bacterial biofilms, likely due to the interaction between the boronic acid moieties and the dextran and/or peptidoglycan domains commonly found in these biofilms. These robust targeting and binding properties may enhance their potential as effective drug carriers in therapeutic applications.

### 2.5 *Lp*‐LNPs Promote the Delivery and Release of Cip to Kill Bacteria and Bacterial Biofilms

**Figure 6:**
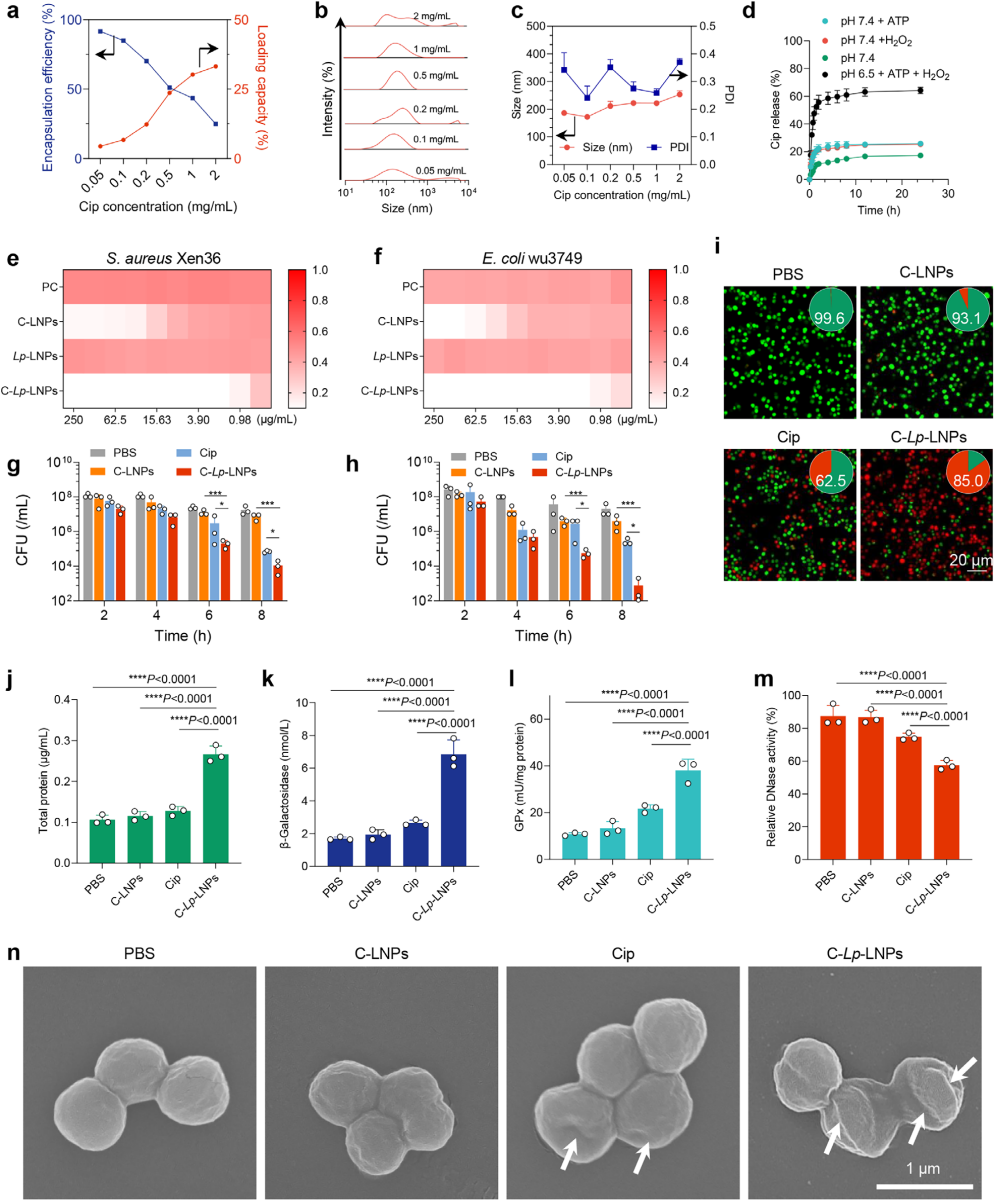
C‐*Lp*‐LNPs efficiently kill bacteria in the planktonic growth state. a) Encapsulation efficiency and loading capacity of Cip in *Lp*‐LNPs with varying Cip feeding concentrations. b) Size distribution of the C‐*Lp*‐LNPs prepared from different Cip loading concentrations, as measured using the DLS method. c) Size and polydispersity index (PDI) of the C‐*Lp*‐LNPs prepared from various Ciploading concentrations, determined via the DLS method. d) Cumulative Cip release from *Lp*‐LNPs composed of 10C lipids after exposure to PB (pH 7.4, 10 mM), 5 mM ATP (PB pH 7.4, 10 mM), 100 µM H_2_O_2_ (PB pH 7.4, 10 mM), and pH 6.5/ATP/H_2_O_2_ (PB, 10 mM), emulating the acidic/oxidative/high ATP microenvironment present in bacterial infections. Data are presented as mean ± standard deviations over three replicates. Data are presented as mean ± standard deviations across three replicates. e) OD_600_ values of *S. aureus* WH^GFP^ suspensions following exposure to various formulations at different concentrations; the MIC concentration was defined as the lowest concentration, resulting in an OD_600_ of less than 0.1. f) OD_600_ values of *E. coli* wu3749 suspensions after exposure to various formulations at different concentrations; the MIC concentration was defined as the lowest drug concentration, yielding an OD_600_ of less than 0.1. g) CFU enumeration of *S. aureus* Xen36 after treatment with different formulations at a Cip concentration of 3.9 µg mL^−1^ over various time intervals. h) CFU enumeration of *E. coli* wu3749 following treatment with different formulations at a Cip concentration of 3.9 µg mL^−1^ over different time intervals. i) CLSM images of *S. aureus* Xen36 after exposure to various formulations at a Cip concentration of 3.9 µg mL^−1^ for 4 h, and bacteria were subjected to live/dead staining, with dead bacteria indicated in red and live bacteria in green. j) Concentration of β‐Galactosidase released from *S. aureus* Xen36 following various treatments for 4 h. k) Concentration of total intracellular protein release from *S. aureus* Xen36 after different treatments for 4 h. l) Concentration of GPx in *S. aureus* Xen36 after various treatments for 4 h. m) Relative DNase activity of *S. aureus* Xen36 after various treatments for 4 h. n) Representative SEM images illustrating the morphology change of *S. aureus* Xen36 after various treatments for 4 h, with white arrows indicating shrinkage of the bacterial cell membrane post‐treatment. Statistical significance was evaluated using one‐way ANOVA with Tukey's multiple comparisons test for (g‐m).

Leveraging the stimuli‐responsiveness of *Lp*‐LNPs, we further investigated the antibiotic loading capabilities of these nanoparticles using Cip as a model antibiotic. As the Cip/*Lp*‐lipid weight ratio increased, the encapsulation efficiency decreased from 91% to 25%, while the loading capacity rose from 4% to 33%. Notably, Cip‐loaded *Lp*‐LNPs (C‐*Lp*‐LNPs) prepared at Cip concentrations of 1 and 0.5 mg mL^−1^ exhibited the optimum size and distribution, whereas C‐*Lp*‐LNPs formulated at other Cip concentrations demonstrated some degree of aggregation (**Figure** **6**a–c). All C‐*Lp*‐LNPs displayed positive surface charges, regardless of the Cip concentration contained within. To identify the optimal Cip‐loaded *Lp*‐LNP formulation, we evaluated drug loading capacity, loading efficiency, and particle size distribution. DLS analysis, including number, volume, and intensity distributions, confirmed minimal aggregation (Figures S19 and S20, Supporting Information) while maintaining high drug encapsulation efficiency (DLE) and drug loading content (DLC), which guided our formulation selection. A Cip loading concentration of 0.5 mg mL^−1^ (Cip/*Lp*‐lipid weight ratio of 1:1) was chosen for further studies. Similar results were observed with Cip loading using *Lp*‐14C‐lipids (Figure S21, Supporting Information). To explore the drug‐loading capacity of our *Lp*‐LNPs for other therapeutics, triclosan and ketoconazole were selected as model drugs. Consistent with the results observed for Cip, the *Lp*‐LNPs demonstrated remarkable loading efficiency, achieving encapsulation efficiencies above 50% and drug loading capacities exceeding 60% at feeding concentrations above 0.5 mg mL^−1^. As the drug loading increased, both the particle size and its distribution widened, while the surface charge remained positively charged (Figures S22 and S23, Supporting Information).

Furthermore, the release of Cip from C‐*Lp*‐LNPs was investigated. At pH 7.4, less than 18% of the loaded Cip was released. The presence of ATP (5 mM) and H_2_O_2_ (100 µM) slightly enhanced the release rate, with 23% of the loaded Cip being released over a 24‐h observation period. Notably, more than 64% of the loaded Cip was released during the same timeframe when C‐*Lp*‐LNPs were exposed to the triple combination of pH 6.5, ATP, and H_2_O_2_ (Figure 6d), indicating that the presence of these three stimuli significantly facilitated Cip release and underscores their potential for treating bacterial infections in an on‐demand manner.

Subsequently, we investigated the bacterial killing efficacy of C‐*Lp*‐LNPs. Neither LNPs nor *Lp*‐LNPs lacking Cip did not affected the growth of drug‐resistant *S. aureus* WH^GFP^ or *Escherichia coli* (*E. coli* wu3749).^[^ ^43^, ^44^ ^]^ The minimum inhibitory concentrations (MICs) of C‐LNPs (in the absence of *Lp*‐lipid) against *S. aureus* and *E. coli* are 39‐fold higher than that of free Cip (Figure 6e,f; Table S1, Supporting Information). In contrast, the MIC values of C‐*Lp*‐LNPs (with *Lp*‐lipid) for *S. aureus* and *E. coli* were one‐fold lower than those of free Cip, indicating that C‐*Lp*‐LNPs were more effective in inhibiting bacterial growth (Figure 6e,f; Table S1, Supporting Information).

In the subsequent bacterial killing studies, we found that C‐*Lp*‐LNPs demonstrated significantly greater efficiency in killing both *S. aureus* and *E. coli* compared to free Cip or C‐LNPs, achieving a minimal bactericidal concentration of 1.95 µg mL^−1^, which was 1‐ and 30‐fold lower than that of free Cip and C‐LNPs, respectively (Table S1, Supporting Information). Similarly, in further kinetic studies of bacterial killing, C‐*Lp*‐LNPs (at Cip concentration of 3.9 µg mL^−1^, 2×MBC) exhibited rapid and more effective killing against *S. aureus* and *E. coli* compared to free Cip or C‐LNPs, with a remarkable 5 to 6 log reduction in the colony‐forming unit (CFU) observed after 8 h (Figure 6g,h). Taken together, our results indicate that C‐*Lp*‐LNPs are more effective in inhibiting bacterial growth and eradicating bacteria, likely due to the presence of the *Lp* lipid domain, which enhances the interaction between the nanoparticles and bacterial cells. Additionally, the controlled release properties of *Lp*‐LNPs enable on‐demand drug release, further contributing to their antibacterial efficacy.

To investigate the mechanisms underlying bacterial killing, we first conducted a live/dead assay. *S. aureus* treated with C‐*Lp*‐LNPs and subjected to live/dead staining exhibited significantly greater red fluorescence compared to all other treatments (Figure 6i), indicating widespread bacterial death and damage to the cell membrane.^[^ ^45^, ^46^ ^]^ Further analysis of intracellular protein release, including total intracellular proteins and specifically β‐galactosidase,^[^ ^47^ ^]^ revealed that C‐*Lp*‐LNPs produced the most substantial release of intracellular proteins (Figure 6j,k). The glutathione peroxidase (GPx) assay demonstrated that bacterial cells treated with C‐*Lp*‐LNPs underwent oxidative stress (Figure 6l), resulting in increased GPx production as a protective response.^[^ ^48^ ^]^ Furthermore, our results showed that C‐*Lp*‐LNP treatment led to a significant increase in intracellular ROS, indicating oxidative stress beyond the activity of ciprofloxacin alone. We observed a marked enhancement in bacterial membrane depolarization after C‐*Lp*‐LNP exposure, which can compromise bacterial viability independent of replication status (Figure S24, Supporting Information). These findings suggest that, in addition to releasing ciprofloxacin, the *Lp*‐LNP carrier system itself contributes to bacterial killing via induction of oxidative stress and membrane disruption. The mechanism of Cip for bacteria‐killing primarily involves the inhibition of bacterial DNA replication, which adversely affects cellular biosynthesis and leads to reduced DNase production.^[^ ^28^, ^49^ ^]^ As a proof‐of‐concept, C‐*Lp*‐LNPs significantly lowered DNase activity compared to other treatment groups (Figure 6m), consistent with the antimicrobial mechanism of Cip. Similar findings were obtained by scanning electron microscopy of bacteria subjected to various treatments, which revealed that C‐*Lp*‐LNPs induced more pronounced damage to the bacterial cell membrane (Figure 6n). Collectively, C‐*Lp*‐LNPs showed a bactericidal mechanism akin to that of free Cip, but they can significantly enhance its killing efficacy.

We subsequently examined the bacterial killing efficacy of *Lp*‐LNPs using *S. aureus* Xen36 biofilms as a model.^[^ ^50^ ^]^ First, 2‐day‐old biofilms were subjected to various treatments and then analyzed through live/dead staining. CLSM images revealed that C‐*Lp*‐LNPs induced the most pronounced red fluorescence, indicating significant damage to bacterial cell membranes (Figure S25a, Supporting Information). In contrast, negligible red fluorescence was observed in biofilms treated with PBS, C‐LNPs, or free Cip. Quantitative analysis of fluorescence intensity corroborated these findings, showing markedly greater red fluorescence in biofilms treated with C‐*Lp*‐LNPs (Figure S25b, Supporting Information).

Furthermore, analysis of biofilm mass, average thickness, and roughness using ImageJ software indicated that C‐*Lp*‐LNPs were notably more effective in eradicating *S. aureus* biofilms compared to all other treatments (Figure S25c–e, Supporting Information). Similar results were obtained from crystal violet staining assays and CFU enumeration, where ≈50% of the biofilms were eliminated, and a 5‐log reduction in CFUs was observed after treatment with C‐*Lp*‐LNPs using a remarkably low Cip concentration of 3.9 µg mL^−1^, approximately threefold more effective than free Cip (Figure S25f,g, Supporting Information). One possible explanation for the loss of biofilm matrix is that Cip released from C‐*Lp*‐LNPs killed the embedded bacteria, weakening their interactions with the matrix. Additionally, the interactions between the *Lp* lipid in C‐*Lp*‐LNPs with dextran and peptidoglycan may compromise the structural integrity of the biofilm matrix, collectively contributing to its disintegration.

### 2.6 C‐*Lp*‐LNPs Alleviate a Peritoneal Infection Caused by *S. aureus*

**Figure 7:**
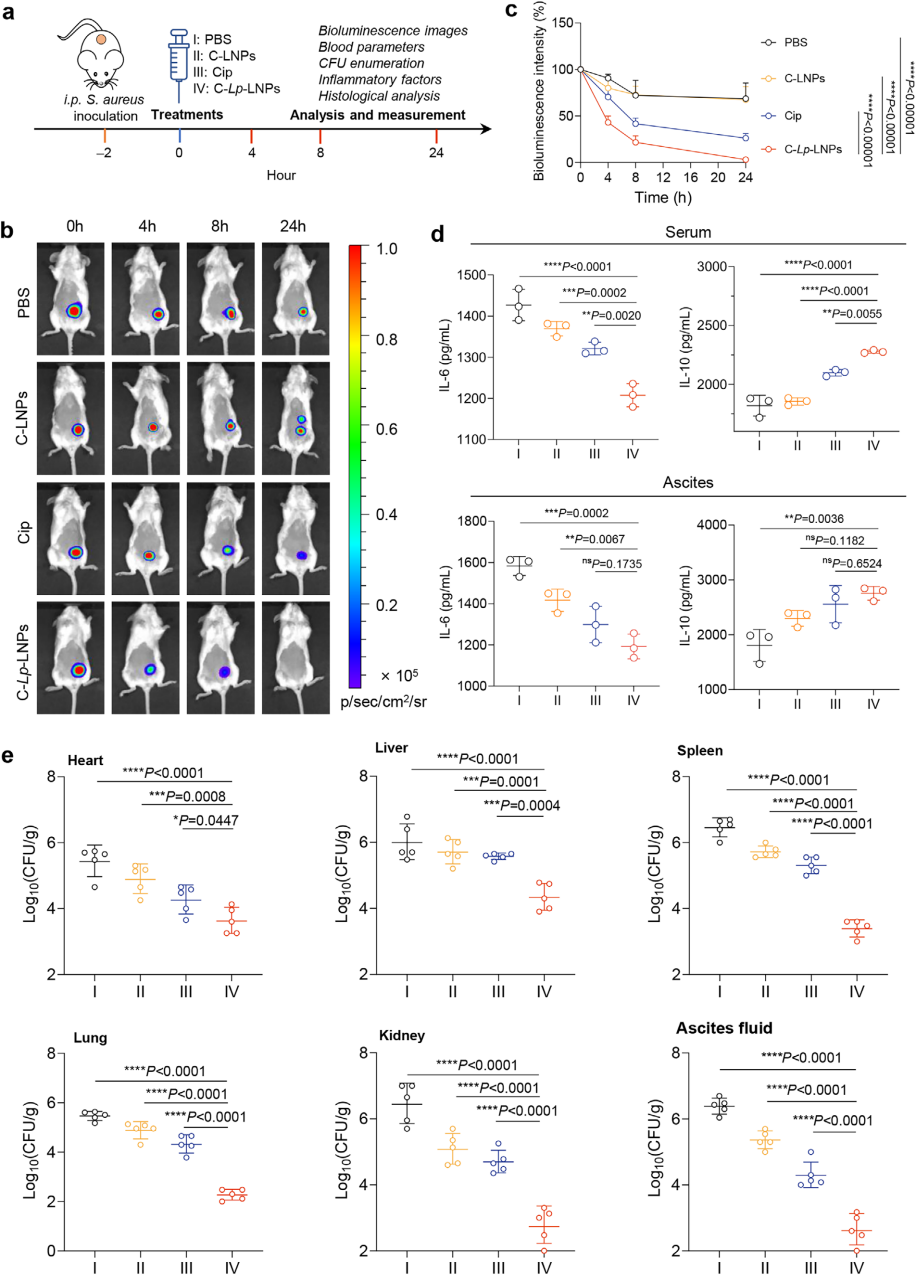
C‐*Lp*‐LNPs effectively eradicate *S. aureus* in a murine peritoneal infection model. a) Workflow illustrating the experimental procedure for the treatment of murine peritoneal infection induced by *S. aureus* Xen36. b) Images depicting changes in bioluminescence at the infection sites of mice receiving various treatments at different time intervals. The Cip dose in all groups was 0.39 µg mouse. For clarity, only one mouse is presented from each group. c) Quantified bioluminescence intensity at the infection sites of mice subjected to various treatments at different time intervals, with the intensity at 0 h set at 100%. d) Expression levels of IL‐6 and IL‐10 in serum and ascites from mice treated for 24 h with different interventions. e) CFU enumeration from major tissues, including heart, liver, spleen, lung, kidney, and ascitic fluid, collected from mice at 24 h post‐treatment. Data are expressed as mean ± standard deviation. Statistical significance was assessed using one‐way ANOVA with Tukey's multiple comparisons test for (d) and (e).

Armed with the promising in vitro physicochemical properties of *Lp*‐LNPs, we proceeded to investigate the in vivo performance of C‐*Lp*‐LNPs and their ability to eradicate bacteria and alleviate inflammation. Toward this goal, we constructed a murine peritoneal infection model utilizing the bioluminescent bacterial strain *S. aureus* Xen36, as described in our previous publications.^[^ ^51^, ^52^ ^]^ The infected mice were subjected to various treatment regimens, which were followed by a variety of assays, as depicted in **Figure** **7**a.

For the assessment of antimicrobial efficacy, we initially captured bioluminescent images of the mice undergoing different treatments, allowing for the non‐invasive evaluation of real‐time antimicrobial effectiveness. Remarkably, C‐*Lp*‐LNPs demonstrated a significant reduction in bioluminescence intensity even with short‐term exposure (for example, just 4 h), outperforming all other treatments by achieving over a 60% reduction in the original bioluminescence intensity within this brief period (Figure 7b,c).

To evaluate the amount of inflammation in the mice, we analyzed the inflammatory factors present in both the ascitic fluid and serum of mice subjected to various treatments. Notably, the pro‐inflammatory cytokine interleukin‐6 was significantly downregulated, while the anti‐inflammatory cytokine interleukin‐10 was upregulated in both serum and ascitic fluid (Figure 7d). In addition, we meticulously examined the number of viable bacteria remaining in various organs and tissues removed from sacrificed mice. The results revealed that C‐*Lp*‐LNPs induced a 2‐5 log reduction in CFU counts in the heart, liver, spleen, lung, and kidney. Most notably, there was an extraordinary reduction of > 5 logs in the ascitic fluid (Figure 7e), underscoring the remarkable antimicrobial efficacy of C‐*Lp*‐LNPs.

These findings indicate a marked alleviation of inflammation, highlighting the therapeutic potential of the treatments administered.^[^ ^53^, ^54^ ^]^ These findings not only confirm the potential of C‐*Lp*‐LNPs as an effective antimicrobial agent but also highlight their capacity to significantly diminish bacterial load and inflammation in vivo.

### 2.7 C‐*Lp*‐LNPs Alleviate a Subcutaneous Infection

**Figure 8:**
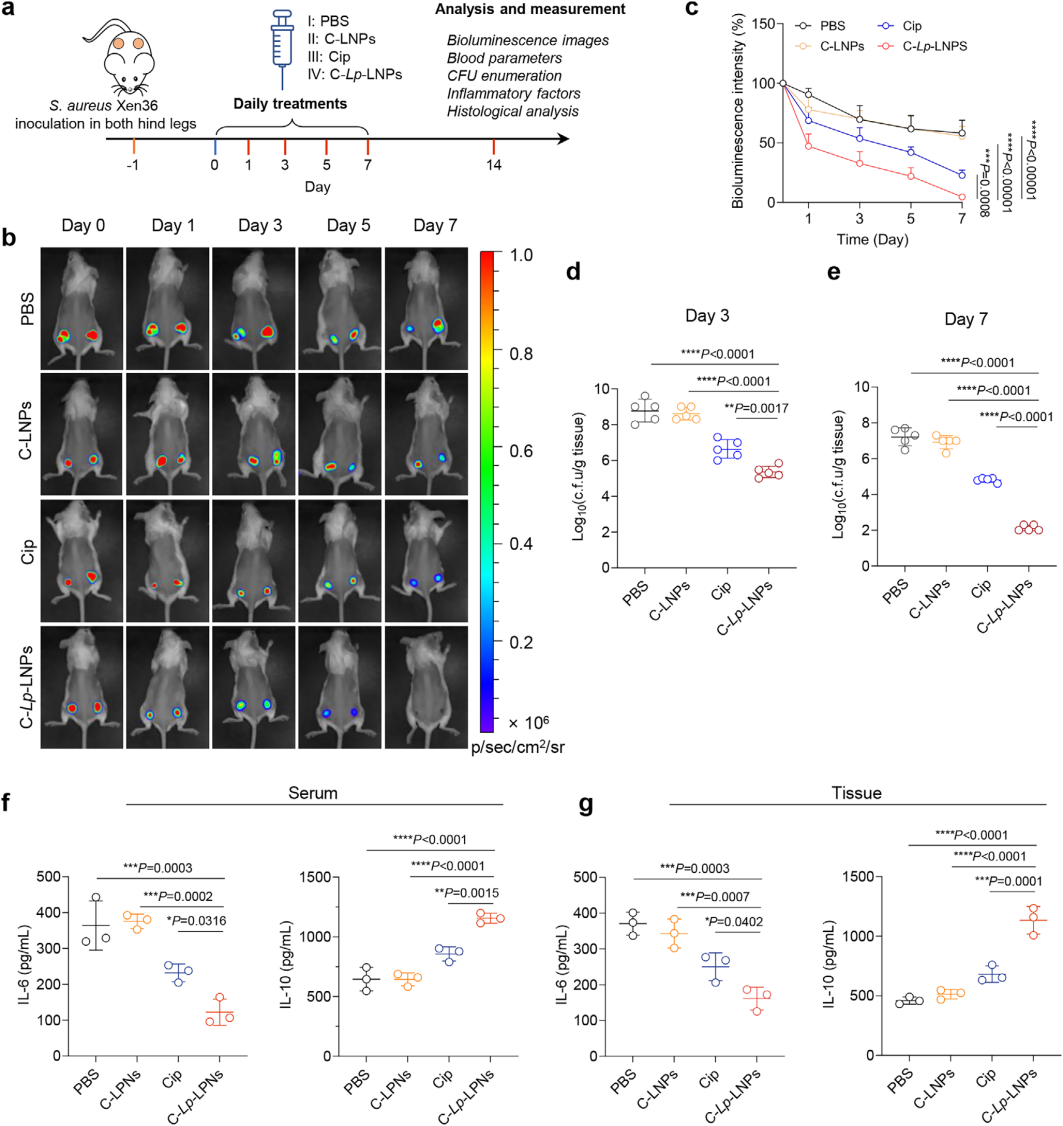
C‐*Lp*‐LNPs effectively eradicate *S. aureus* in a murine subcutaneous infection model. a) Workflow illustrating the experimental procedure for the treatment of murine subcutaneous infection induced by *S. aureus* Xen36. b) Images depicting changes in bioluminescence at the infection sites of mice receiving various treatments at different time intervals. The Cip dose in all groups was 0.3 µg/mouse. For clarity, only one mouse is presented from each group. c) Quantified bioluminescence intensity at the infection sites of mice subjected to various treatments at different time intervals, with the intensity at 0 h set at 100%. d, e) CFU enumeration from the infected tissue collected from mice on days 3 (d) and 7(e) post‐treatment. f, g) Expression levels of IL‐6 and IL‐10 in serum f) and infected tissue (g) from mice treated at day 7 with different interventions. Data are expressed as mean ± standard deviation. Statistical significance was assessed using one‐way ANOVA with Tukey's multiple comparisons test for (d‐g).

To evaluate the therapeutic potential of C‐*Lp*‐LNPs in eradicating long‐term infections, we employed a murine subcutaneous infection model using bioluminescent *S. aureus* Xen36 (**Figure** **8**a). Remarkably, C‐*Lp*‐LNPs significantly reduced the bioluminescence intensity as early as day 1 post‐treatment, outperforming all other treatment groups. By day 7, mice treated with C‐*Lp*‐LNPs exhibited only marginal bioluminescence signals, in stark contrast to the persistent and pronounced signals observed in the groups receiving free Cip, Cip‐loaded LNPs, or untreated controls (Figure 8b,c).

Quantitative bacterial enumeration further confirmed the superior antibacterial activity of C‐*Lp*‐LNPs. On day 3 post‐treatment, C‐*Lp*‐LNPs achieved a substantial 3.5‐log reduction in CFU counts—significantly more effective than either free Cip or Cip‐LNPs. By day 7, the CFU reduction reached an impressive 5‐log scale, indicating near‐complete bacterial eradication and demonstrating a two orders of magnitude enhancement in bacterial clearance compared to free Cip (Figure 8d,e). This enhanced performance likely results from the targeted delivery and improved retention of C‐*Lp*‐LNPs at the infection site, facilitating more efficient drug accumulation and bacterial uptake.

In addition to their potent antibacterial effects, C‐*Lp*‐LNPs also modulated the host immune response. Treatment with C‐*Lp*‐LNPs led to a significant downregulation of the proinflammatory cytokine IL‐6, while concurrently upregulating the anti‐inflammatory cytokine IL‐10 in both serum and infected tissue (Figure 8f,g). These changes suggest that C‐*Lp*‐LNPs not only promote bacterial clearance but also contribute to the resolution of inflammation, highlighting their dual therapeutic benefit in managing persistent infections.

### 2.8 Biocompatibility Assessment

To thoroughly evaluate the biocompatibility of C‐*Lp*‐LNPs, we started by assessing their cytotoxicity using murine fibroblast L929 cells as a model system.^[^ ^44^ ^]^ The results demonstrated that the cells maintained over 85% of their original viability when exposed to C‐*Lp*‐LNPs at concentrations of up to 250 µg mL^−1^ (Figure S26, Supporting Information), indicating a favorable safety profile. Subsequently, we proceeded to measure the hemolysis rate at various concentrations. Remarkably, we found that the hemolysis rate of C‐*Lp*‐LNPs remained consistently below 5%, even at a remarkably high concentration of 250 µg mL^−1^ (Figure S27, Supporting Information). This finding underscores the biocompatibility of C‐*Lp*‐LNPs and their potential for safe therapeutic applications.

By carefully monitoring hematological parameters before and after the administration of C‐*Lp*‐LNPs, we were able to assess their biocompatibility and identify any potential alterations in blood composition resulting from treatment. Notably, our results demonstrated that none of the formulations caused significant hematological changes postadministration, indicating a favorable safety profile for C‐*Lp*‐LNPs (Figure S28, Supporting Information). Consistent findings were observed in the long‐term infection model following various treatments. On both days 3 and 7, mice treated with C‐*Lp*‐LNPs exhibited no significant changes in hematological indices (Figure S29 and S30, Supporting Information). In contrast, mice receiving PBS or C‐LNPs showed a marked increase in white blood cells and neutrophils on day 3, indicative of an ongoing inflammatory response. These results further support the therapeutic potential of C‐*Lp*‐LNPs, not only in eradicating infection but also in minimizing systemic inflammatory side effects.

These results were further confirmed by histological staining of tissue from major organs removed from mice subjected to various treatments (Figures S31–S33, Supporting Information). The histological evaluations revealed no notable pathological alterations on days 1, 7, and 14 post‐treatment, thereby reinforcing the safety and biocompatibility of C‐*Lp*‐LNPs. This comprehensive approach not only highlighted the importance of assessing both blood parameters and tissue structure but also underscored the potential of C‐*Lp*‐LNPs as a viable therapeutic option. The absence of significant hematological changes and the preservation of tissue architecture suggest that C‐*Lp*‐LNPs may be safely employed in clinical applications, paving the way for future investigations into their efficacy and therapeutic potential in treating bacterial infections and enhancing conventional antibiotic therapy.

## 3 Conclusion

In conclusion, to enable the integration of both targeting and stimuli‐responsive properties within a single small molecule for enhanced drug delivery, we developed a novel class of *Lp*‐lipids. These lipids are composed of phenylboronic acid as a Lewis acid and amine as a Lewis base, strategically designed to combine the benefits of both chemical functionalities. The resulting *Lp*‐lipids were then utilized to fabricate antibiotic‐loaded LNPs aimed at the treatment of bacterial infections. In particular, when targeting bacterial biofilms, the phenylboronic acid group selectively binds to biofilm structures through interactions with diol groups in dextran or peptidoglycan. This binding process is crucial for effective biofilm targeting, which is notoriously difficult in antimicrobial applications. Furthermore, the amine group in the *Lp*‐lipids undergoes protonation in acidic environments, thereby facilitating electrostatic interactions with bacterial surfaces and biofilms. This electrostatic attraction significantly enhances the targeting of bacteria and biofilms, optimizing the delivery of the therapeutic payload.

For stimuli‐responsive drug release, the amine domain of the *Lp*‐lipids responds to the acidic microenvironment typical of infected tissues, leading to changes in the lipid structure that promote drug release. Additionally, the phenylboronic acid domain is responsive to H_2_O_2_ and ATP, both of which are important signals in the infected environment. Collectively, these stimuli induce a significant alteration in the hydrophobicity of the *Lp*‐lipids, triggering the release of the encapsulated Cip, a potent antibiotic, thereby effectively eradicating the embedded bacteria within the biofilms. The unique combination of targeting and stimuli‐responsive properties endowed by our *Lp*‐lipids resulted in the successful creation of C‐*Lp*‐LNPs, which exhibited excellent biofilm‐targeting capabilities and bactericidal activity against both *S. aureus* and *E. coli*. In in vivo models, particularly in a peritoneal infection induced by *S. aureus*, our C‐*Lp*‐LNPs demonstrated an ability to efficiently eliminate the infecting bacteria while simultaneously alleviating the associated inflammatory response, all without causing harm to surrounding healthy tissues or cells.

Overall, the *Lp*‐lipids are a promising class of molecules that effectively combine targeting and stimuli‐responsiveness within a single entity, offering a significant advance in the field of targeted drug delivery. Moreover, the simplicity of their synthesis and their versatility in application provide immense potential for the development of advanced drug delivery systems. Our ongoing research is exploring the broader applications of these *Lp*‐lipids in the treatment of other diseases, including tumors and inflammatory conditions, which are currently being investigated in our laboratory.

## 4 Experimental Section

### Animals

Female ICR mice (6 weeks, 25.0–27.0 g) were purchased from Zhejiang Vital River Laboratory Animal Technology Co., Ltd and housed in an SPF room. The animal experimental protocols were reviewed and approved by the Institutional Animal Care and Use Committee, Wenzhou Institute, University of Chinese Academy of Sciences (No. WIUCAS24040306).

### Statistical Analysis

Statistically significant differences between two specific groups were analyzed using the Student's *t*‐test. The statistically significant differences between three or more groups were analyzed by one‐way ANOVA. *ns, p *≥ 0.05, * *p *< 0.05, ** *p *< 0.01, *** *p *< 0.001, and **** *p *< 0.0001.

## Conflict of Interest

The authors declare no conflict of interest.

## Supporting information



Supporting Information

## Data Availability

The data that support the findings of this study are available from the corresponding author upon reasonable request.
