# Supplementary material for: A Lipid with Lewis Pair‐Mediated Targeting and Multiple Stimuli‐Responsive Delivery of Antibiotics for Bacterial Infections
Source: Adv Sci (Weinh). 2025 Aug 24;12(43):e07407. doi: 10.1002/advs.202507407 (PMC12631898; doi:10.1002/advs.202507407)
Supplement: Supplementary file 1 — Supporting Information [file ADVS-12-e07407-s001.docx]

Supporting Information

**A Lipid with Lewis Pair-Mediated Targeting and Multiple Stimuli-Responsive Delivery of Antibiotics for Bacterial Infections**

*Xiaojian Yan#, Lei Hua#, Hongping Wan#, Yong Liu*, Tao Jin, Yicen Ge, Michael R Hamblin, Mahdi Karimi*, Xinghong Zhao*, Yan Hu*, Linqi Shi, Yuanfeng Li**

Contents

[Materials and methods 3](#_Toc200466635)

[*Chemicals and materials* 3](#_Toc200466636)

[*Bacterial strains* 4](#_Toc200466637)

[*Cells* 4](#_Toc200466638)

[*Animals* 4](#_Toc200466639)

[*Characterizations* 4](#_Toc200466640)

[*Synthesis of Lp-lipids* 4](#_Toc200466641)

[*Preparation of the LNPs and Lp-LNPs* 5](#_Toc200466642)

[*Responsiveness of LNPs* 6](#_Toc200466643)

[*Drug Loading and Release* 7](#_Toc200466644)

[*Responsive Drug Release* 8](#_Toc200466645)

[*Molecular Dynamics Simulation Methods* 8](#_Toc200466646)

[*Interaction with Bacteria* 9](#_Toc200466647)

[*Binding of Lp-LNPs to Bacterial Biofilms* 10](#_Toc200466648)

[*Antibacterial Effects In Vitro* 10](#_Toc200466649)

[*Eradication of Mature Biofilms* 12](#_Toc200466650)

[*Cytotoxicity Assessment* 13](#_Toc200466651)

[*Hemolysis Assessment* 13](#_Toc200466652)

[*Acute Peritonitis Model* 13](#_Toc200466653)

[*Chronic Subcutaneous Infection Model* 14](#_Toc200466654)

[*Statistical Analysis* 14](#_Toc200466655)

[Supplementary Figures 14](#_Toc200466656)

[Figure S1. 14](#_Toc200466657)

[Figure S2. 15](#_Toc200466658)

[Figure S3. 15](#_Toc200466659)

[Figure S4. 16](#_Toc200466660)

[Figure S5. 16](#_Toc200466661)

[Figure S6. 17](#_Toc200466662)

[Figure S7. 17](#_Toc200466663)

[Figure S8. 18](#_Toc200466664)

[Figure S9. 18](#_Toc200466665)

[Figure S10. 18](#_Toc200466666)

[Figure S11. 19](#_Toc200466667)

[Figure S12. 19](#_Toc200466668)

[Figure S13. 19](#_Toc200466669)

[Figure S14. 20](#_Toc200466670)

[Figure S15. 21](#_Toc200466671)

[Figure S16. 21](#_Toc200466672)

[Figure S17. 20](#_Toc200466673)

[Figure S18. 20](#_Toc200466674)

[Figure S19. 21](#_Toc200466675)

[Figure S20. 22](#_Toc200466676)

[Figure S21. 22](#_Toc200466677)

[Figure S22. 23](#_Toc200466678)

[Figure S23. 22](#_Toc200466679)

[Figure S24. 23](#_Toc200466680)

[Figure S25. 24](#_Toc200466681)

[Figure S26. 24](#_Toc200466682)

[Figure S27. 25](#_Toc200466683)

[Figure S28. 26](#_Toc200466684)

[Figure S28. 27](#_Toc200466685)

[Figure S29. 28](#_Toc200466686)

[Figure S30. 29](#_Toc200466687)

[Figure S31. 30](#_Toc200466688)

[Figure S32. 31](#_Toc200466689)

[Table S1 32](#_Toc200466690)

# Materials and methods

## *Chemicals and materials*

Diphenyl phosphite (80%), 1-decanol (98%), pyridine (99.9%), N,N-dimethylethane-1,2-diamine (98%) were purchased from Energy Chemical (Shanghai, China). (4-formylphenyl)boronic acid (98%) was purchased from Macklin (Shanghai, China). L-α-phosphatidylcholine (Egg PC,95%) and cationic lipid 1,2-dioleoyl-3-trimethylammonium propane (DOTAP) were purchased from Aladdin (Shanghai, China). Ciprofloxacin (Cip) was purchased from Bidepharm (China). Chloroform (CHCl_3_) was purchased from J&K Scientific (Beijing, China). SYTO™ 9, propidium iodide (PI), and cell counting kit-8 (CCK-8) were purchased from Beyotime (Shanghai, China). DiR Iodide was purchased from Yeasen Biotechnology (Shanghai, China). Adenosine triphosphate (ATP, 98%), peptidoglycan (98%) and Nile Red (95%) were purchased from Macklin (Shanghai, China). Glypican-3 and dextran T10 were purchased from Yuanye Bio-Technology Co., Ltd (Shanghai, China). Dulbecco's Modified Eagle Medium (DMEM), penicillin-streptomycin (PS) solution, phosphate-buffered saline (PBS), and fetal bovine serum (FBS) for cell culture were purchased from Gibco Life Technologies, Inc. (Grand Island, NY, USA). Mouse IL-6 and IL-10 ELISA kits were purchased from iCell Bioscience Inc. (Shanghai, China). BCA Protein Assay Kit (BCA), DNase Activity Fluorometric Assay Kit and Glutathione Reductase Assay Kit with DTNB were purchased from Beyotime (Shanghai, China). β-Galactosidase(β-GAL) Activity Assay Kit was purchased from Solarbio (Beijing, China). All solvents and reagents were commercially available and used without further purification unless noted otherwise.

## *Bacterial strains*

Two staphylococcal strains, *S. aureus* Xen36 (PerkinElmer Inc., Waltham, MA, USA) and *S. aureus* WH^GFP^, as well as *Escherichia coli* WU3749 (clinical isolate, kindly provided by Zhou Lab at the First Affiliated Hospital of Wenzhou Medical University), were utilized in this study. For experiments, *S. aureus* was cultured on tryptic soy broth (TSB) agar plates, and *E. coli* was cultured on lysogeny broth (LB) plates. A single colony of *S. aureus* was inoculated in 10 mL TSB (OXOID, Basingstoke, UK) (or LB for *E. coli*) and incubated for 24 hours at 37 °C with shaking. This culture was used to inoculate a 200 mL main culture at a 1:20 dilution and grown for 16 hours. Bacterial cultures were harvested by centrifugation at 5000 *g* for 5 minutes, washed twice in PBS (pH 7.4), resuspended in 10 mL of PBS, and the bacterial concentration was determined using a Bürker-Türk counting chamber (Marienfeld, Germany).

## *Cells*

Mouse fibroblast (L929) cells were involved in this study. L929 cells were cultured in a DMEM containing 10% FBS and 1% penicillin-streptomycin solution at 37 °C with 5% CO_2_. Human embryonic kidney (293T) cells were purchased from Thermo Fisher Scientific, Inc. (Carlsbad, USA). L929 were cultured in DMEM medium, supplemented with 10% fetal bovine serum (FBS, Gibco, Shanghai, China), penicillin (100 U/mL, Genview, Beijing, China) and streptomycin (100 μg/mL, Solarbio, Beijing, China).

## *Animals*

Female ICR mice (6 weeks, 25.0-27.0 g) were purchased from Zhejiang Vital River Laboratory Animal Technology Co., Ltd and housed in an SPF room. The animal experimental protocols were reviewed and approved by the Institutional Animal Care and Use Committee, Wenzhou Institute, University of Chinese Academy of Sciences (No. WIUCAS24040306).

## *Characterizations*

The size and zeta potential of nanoparticles were determined using a Zetasizer Nano ZEN3600 instrument (Malvern, UK). The morphology of the NPs was examined using a Talos F200S transmission electron microscope (Thermo Scientific, US). Matrix-assisted laser desorption/ionization-time of flight (MALDI-TOF) mass spectrometry (MS) analysis was carried out using an AutoFlex Max Mass Spectrometer (Bruker, Germany).

## *Synthesis of Lp-lipids*

Firstly, a mixture of diphenyl phosphite (5.0 mmol, 1.0 equiv), 1-decanol (10.0 mmol 2.0 equiv), and pyridine (0.5 mL) was placed in a distillation apparatus at 140 °C under reduced pressure (4.10−2 Mbar) for 4 h. Pyridine and phenol were then removed by distillation. Di-decanyl phosphonate (> 94% yield) was obtained from the residue remaining after distillation.

For the synthesis of *Lp*-lipids, N,N-dimethylethane-1,2-diamine (88.10 mg, 1 mmol, 1.0 equiv), and (4-formylphenyl)boronic acid (149.94 mg, 1 mmol, 1.0 equiv) were dissolved in methanol (3 mL) and heated to 70 °C for 3 h under nitrogen. Then, the mixture was evaporated at 30 °C, and di-decanyl phosphonate (1 mmol, 1.0 equiv) was added, followed by 1.5 h of stirring at 100 °C. The crude product was purified by chromatography on a silica gel column (CH_2_Cl_2_ as eluent) to yield the *Lp*-containing α-aminophosphonate.

*Lp*-10C-lipid, brown solid, yield: 70.1%: ^1^H NMR (400 MHz, CDCl_3_) δ 7.88 (d, *J* = 8.1 Hz, 1H), 7.41 (dd, *J* = 8.2, 2.3 Hz, 1H), 4.12 – 3.93 (m, 2H), 3.90 – 3.78 (m, 1H), 3.78 – 3.63 (m, 1H), 2.80 – 2.58 (m, 1H), 2.56 – 2.42 (m, 1H), 2.22 (s, 3H), 1.61 (dd, *J* = 14.2, 7.0 Hz, 1H), 1.49 (dd, *J* = 13.5, 6.8 Hz, 1H), 1.33 – 1.24 (m, 7H), 1.21 (s, 8H), 0.94 – 0.85 (m, 3H), 0.85 (dd, *J* = 7.0, 4.3 Hz, 3H). ^13^C NMR (101 MHz, CDCl_3_) δ 129.45, 115.70, 67.07, 66.76, 52.41, 46.59, 31.88, 30.57, 29.55, 29.31, 29.22, 29.17, 25.49, 25.43, 22.67, 14.10, 11.30, 1.00. ^31^P NMR (162 MHz, CDCl_3_) δ 24.03.

^^

*Lp*-14C-lipid, brown solid, yield: 62.5%: ^1^H NMR (400 MHz, CDCl_3_) δ 7.39 (d, *J* = 8.2 Hz, 1H), 7.33 (t, *J* = 7.2 Hz, 1H), 3.97 (dd, *J* = 7.3, 2.2 Hz, 1H), 3.86 (dt, *J* = 13.4, 6.7 Hz, 1H), 3.78 – 3.64 (m, 1H), 2.88 – 2.82 (m, 1H), 2.79 – 2.72 (m, 1H), 2.53 (s, 1H), 1.59 (t, *J* = 7.0 Hz, 1H), 1.44 (t, *J* = 6.7 Hz, 1H), 1.21 (d, *J* = 23.1 Hz, 7H), 0.87 (t, *J* = 6.8 Hz, 1H). ^13^C NMR (100 MHz, CDCl_3_) δ 128.56, 128.50, 128.42, 66.86, 61.94, 58.45, 45.03, 31.94, 30.61, 29.72, 29.68, 29.61, 29.53, 29.38, 29.21, 29.16, 25.47, 25.38, 22.71, 14.13, 1.03. ^31^P NMR (162 MHz, CDCl_3_) δ 23.19.

## *Preparation of the LNPs and Lp-LNPs*

Firstly, stock solutions of egg PC (1 mg/mL) and *Lp*-lipid (1 mg/mL) in chloroform were prepared. For nanoparticle preparation, 1 mL of the PC solution and 100 µL of the *Lp*-lipid solution (PC/*Lp*-lipid weight ratio = 10:1) were pipetted into a flask and mixed thoroughly to ensure complete dissolution. The mixture was then subjected to rotary evaporation at 50 °C to remove the chloroform. Following this, the round-bottom flask was placed in a vacuum oven at 50 °C for 1 hour to further dry the sample. Subsequently, 1 mL of phosphate buffer (pH 7.4, 10 mM) was added with ultrasonic mixing. The flask was sealed with a stopper, and the sample underwent an alternating cycle of ultrasonication (40kHz, 480 W, Scientz-650E, China) and vortexing for 30 minutes. Thereafter, the mixture was passed through an extruder fitted with a 0.4 μm membrane, undergoing a total of 19 extrusion cycles. For the control group, the same procedure was followed, except that no *Lp*-lipid or DOTAP was employed.

## *Responsiveness of LNPs*

(a) ATP treatment. 100 μL of C-LNPs or C-*Lp*-LNPs (4 mg/mL) was mixed with 890 μL of pH 7.4 phosphate buffer (PB, 0.1M) and 10 μL of ATP (500 mM in water). The final NP concentration was 400 µg/mL and ATP concentration was 5 mM.

(b) H_2_O_2_ treatment. 100 μL of C-LNPs or C-*Lp*-LNPs (4 mg/mL) was mixed with 890 μL of pH 7.4 phosphate buffer (PB, 0.1M) and 10 μL of H_2_O_2_ (10 mM). The final NP concentration was 400 µg/mL and H_2_O_2_ concentration was 100 µM.

(c) Acidity treatment. 100 μL of C-LNPs or C-*Lp*-LNPs (4 mg/mL) was mixed with 900 μL of phosphate buffer (PB, 0.1M) with pH ranging from 5.5 to 7.4. The final NP concentration was 400 µg/mL.

(d) Triple stimuli (pH 6.5/ATP/H_2_O_2_) treatment. 100 μL of C-LNPs or C-*Lp*-LNPs (4 mg/mL) was mixed with 880 μL of phosphate buffer (PB, pH 6.5, 0.1M), 10 μL of H_2_O_2_ (10 mM), and 10 μL of ATP (500 mM in water). The final NP concentration was 400 µg/mL, H_2_O_2_ concentration was 100 µM, and ATP concentration was 5 mM.

(e) Glucose treatment. A 100 μL aliquot of Lp-LNPs (4 mg/mL) was mixed with 890 μL of phosphate buffer (PB, 0.1 M, pH 7.4) and 10 μL of glucose solution (10 mg/mL or 20 mg/mL in water). The final concentrations were 400 μg/mL for Lp-LNPs and 0.1 mg/mL or 0.2 mg/mL for glucose. The mixtures were incubated at 37 °C for 1, 2, and 4 hours. After incubation, the particle size and zeta potential were measured using a Zetasizer Nano ZEN3600 instrument (Malvern, UK).

**Characterizations of NPs**

(a) *Size and zeta potential measurement:* The mixtures were incubated at 37 °C for 2 hours, after which the particle size and zeta potential were determined on a Zetasizer Nano ZEN3600 instrument (Malvern, UK).

(b) *TEM Measurement:* To prepare for transmission electron microscopy (TEM) analysis, 100 μL of C-LNPs or C-*Lp*-LNPs (1 mg/mL) were added to 880 μL of phosphate buffer (PB) with a pH of 6.5. Subsequently, 10 μL of ATP (500 mM) and 10 μL of H_2_O_2_ (10 mM) were added, and the mixture was co-incubated in 1.5 mL EP tubes for 8 hours. After incubation, 10 μL of the solution was placed onto a formvar-stabilized carbon support film for TEM analysis on a Talos F200S transmission electron microscope (Thermo Scientific, US).

(c) *Atomic Force Microscopy (AFM)* *measurement:* AFM measurements of the nanoparticles with different stimuli were performed on a Bio-atomic force microscope, Bruker JPK Nanowizard V (Bruker, Germany), equipped with an SAA-SPH-5UM cantilever with a cylindrical tip. Nanoparticles prepared in phosphate buffer (10 mM, pH 7.4) were subjected to various stimuli to achieve the desired conditions (pH 7.4; pH 7.4 with 5 mM ATP; pH 7.4 with 100 µM H_2_O_2_; pH 6.5; pH 6.5 with 5 mM ATP and 100 µM H_2_O_2_). The post-stimulation nanoparticles were immediately applied to a silicon wafer for 0.5 hours. After that, the silicon wafer was washed with PBS five times to remove the unattached nanoparticles. The cantilever was carefully positioned on the silicon wafer, and peak force tapping mode was used for quantitative imaging. The deﬂection sensitivity was 24.4 nm/V, and the spring constant was 3.0 N/m, with a duration 6.7 ms, and a constant approach and retraction speed 45 μm/s. Images of 128 × 128 pixels were recorded on areas of 30 μm × 30 μm surface. Image analysis was carried out by JPKSM Data Processing Version 8.0.99 by determining the height of several well-isolated particles from the corresponding images.

## *Drug Loading and Release*

First, the absorption spectra of free Cip in phosphate buffer (PB, pH 7.4, 10 mM) at varying concentrations were measured using a UV-vis spectrometer (UV-1900i, Shimadzu, Japan). A calibration curve was constructed using the absorption peak of free Cip at 280 nm. The standard curve equation is:

A_280_ = 93.174 × *C* - 0.0534 (R² = 0.9998, at 280 nm),

where A_280_ represents the absorbance at 280 nm, and *C* denotes the concentration of Cip.

To investigate the encapsulation of Cip within LNPs, stock solutions of egg phosphatidylcholine (PC) (1 mg/mL) and *Lp*-lipid (1 mg/mL) in chloroform were prepared. For nanoparticle formation, 900 µL of the PC solution and 100 µL of the *Lp*-lipid solution were pipetted into a flask and thoroughly mixed to ensure complete dissolution. The mixture was subsequently subjected to rotary evaporation at 50 °C to remove the chloroform. Following this, the round-bottom flask was placed in a vacuum oven at 50 °C for 1 hour to further dry the sample. Next, 1 mL of milli-Q water and Cip (1 mg/mL in milli-Q water) were added at various weight ratios of lipid to Cip, ranging from 0.05 to 2. The flask was sealed with a stopper, and the sample underwent alternating cycles of ultrasonication (40 KHz, 480 W, Scientz-650E, China) and vortexing for 30 minutes. Thereafter, the mixture was passed through an Avanti mini extruder fitted with a 0.4 μm membrane, undergoing a total of 19 extrusion cycles. The unencapsulated Cip was then removed by centrifugation at 20,000 rpm for 1 h. The encapsulation efficiency (*EE*) and loading capacity (*LC*) of the drug were determined as follows:

$$\begin{aligned} &EE(\%)=(1-\frac{C_{s}\times V_{s}}{W_{0}})\times100\% (1) \\ &LC(\%)=\frac{W_{0}\times\mathrm{EE}\left( \% \right)}{W_{0}\times\mathrm{EE}\left( \% \right)+W_{P}}\times100\% (2) \end{aligned}$$

Where *C*_s_ and *V*_s_ are Cip concentration and volume in the supernatant after centrifugation, respectively. *W*_0_ and *W*_p_ represent the mass of Cip and *Lp*-LNPs added to the system, respectively.

To investigate the encapsulation of triclosan and ketoconazole within LNPs, a method similar to that used for Cip loading was employed. Stock solutions of egg phosphatidylcholine (PC, 1 mg/mL), *pba*-lipid (1 mg/mL), triclosan (5 mg/mL), and ketoconazole (5 mg/mL) in chloroform were prepared. For nanoparticle formation, 1 mL of PC solution, 100 µL of *pba*-lipid, and varying volumes of triclosan or ketoconazole (20, 40, 100, 200, or 400 µL) were pipetted into a flask and thoroughly mixed to ensure complete dissolution. The mixture was then subjected to rotary evaporation at 50 °C to remove chloroform, followed by further drying in a vacuum oven at 50 °C for 1 hour. Subsequently, 1 mL of Milli-Q water was added, and the sample was processed by ultrasonication and extrusion. Unencapsulated triclosan or ketoconazole was removed via centrifugation. The EE and LC were calculated using the formulas described earlier. Drug concentrations were determined by UV-Vis spectroscopy, with the following standard curves:

Triclosan: A_281_ = 0.0699 × C - 0.003 (R^2^ = 0.9995, at 281 nm);

Ketoconazole: A_295_ = 0.0499 × C - 0.0074 (R^2^ = 0.9838, at 295 nm).

Finally, the particle size and zeta potential of the drug-loaded *Lp*-LNPs were measured using a Zetasizer Nano ZEN3600 instrument (Malvern, UK).

## *Responsive Drug Release*

To investigate the release of Cip from C-*Lp*-LNPs, a suspension of C-*Lp*-LNPs (1 mL, 3 mg/mL) was placed in a dialysis bag (cut-off molecular weight: 500 Da) and immersed in solutions (20 mL) of phosphate buffer (10 mM, pH 7.4 or 6.5), with or without 100 µM H₂O₂ and 5 mM ATP, at 37 °C to facilitate Cip release. At various time intervals, aliquots (3.0 mL) of the dialysis solution were collected and replaced with fresh dialysis fluid (3.0 mL). The absorbance of the sample at 280 nm was recorded using a UV-vis spectrometer.

## *Molecular Dynamics Simulation Methods*

To gain a mechanistic understanding of the experimental findings, we performed all-atom simulations to investigate the effect of introducing *Lp*-lipid and *Lp*-lipid+ATP molecules on the structural stability of the egg PC phospholipid bilayer membrane. The egg PC bilayer membrane model was generated using the CHARMM-GUI tool, with a simulated system size of 10 nanometers by 10 nanometers and a total of 338 POPC molecules. To mimic a realistic biological environment, the system was solvated with TIP3P water molecules, and sodium and chloride ions were included at a concentration of 0.1 M for electrical neutrality. In the hybrid systems, 30 egg PC molecules were randomly substituted with *Lp*-lipid or *Lp*-lipid+ATP molecules using the Gromacs insert-molecules tool. The molecular models and topological information for the *Lp*-lipid and *Lp*-lipid+ATP were obtained from the ATB tool.

The simulation was carried out in the NPT ensemble using Gromacs version 2021.4, with the Nose-Hoover thermostat maintaining the temperature at 303.15 K and the semi-isotropic Parrinello-Rahman barostat regulating the pressure at 1 bar. Following a comprehensive relaxation period, the simulation ran for 100 nanoseconds to capture the ensemble average properties of the stabilized membrane. Subsequently, the results were analyzed using VMD software to provide both visual and quantitative insights.

## *Interaction with Bacteria*

*CLSM:* Initially, Nile red-loaded LNPs and *Lp*-LNPs were prepared by adding 10 µg of Nile red to a PC solution before it was subjected to rotary evaporation. The other processes were kept the same as the LNPs and *Lp*-LNPs preparation. The unloaded Nile red was removed by centrifugation. To investigate the interactions of *S. aureus* WH^GFP^ with LNPs or *Lp*-LNPs, *S. aureus* WH^GFP^ (10⁹ bacteria/mL, 100 µL) was added to a confocal dish (35 mm, NEST) and allowed to stand for 1 hour to facilitate bacterial attachment. Subsequently, the bacterial suspension was removed and washed three times with PBS (100 µL each). Thereafter, the Nile red-loaded LNPs or *Lp*-LNPs were introduced to the confocal dish and observed using a Nikon A-1 confocal microscope (Nikon, Japan). The fluorescence intensity of Nile red surrounding a single bacterium was quantified using ImageJ software (version 1.8.0).

*Flow cytometry:* For flow cytometry, DiR-LNPs and DiR-*Lp*-LNPs were prepared by mixing 200 μL of LNPs or *Lp*-LNPs (2 mg/mL) with DiR (20 µg/mL, Yeasen Biotechnology, Shanghai, China) for 30 minutes. The unloaded DiR was removed by centrifugation. Subsequently, 1 mL of *S. aureus* WH^GFP^ (10^9^ bacteria/mL) was added and incubated at 37 °C for 2 hours. Finally, flow cytometry (Beckman CytoFlex, California, USA) was conducted to evaluate the interaction between the LNPs and the bacteria.

Interaction studies of Lp-LNPs with cells and bacteria: Initially, *Lp*-LNPs^DiR^ were prepared by mixing *Lp*-LNPs (2 mg/mL) with DiR (20 µg/mL) for 30 minutes. The unstained DiR was removed by centrifugation. *S. aureus* WH^GFP^ (1×10⁹ CFU/mL) was stained with SYTO9 dye according to the manufacturer’s protocol, and the excess SYTO9 dye was removed by centrifugation. The 293T cell were resuspended in 24-well plates (10^5^ cell/mL, 2 mL) and stained with Hoechst dye according to the manufacturer’s protocol, and the residual Hoechst dye was removed by centrifugation.

To investigate the interactions of *Lp*-LNPs^DiR^ with cell and bacteria, the *Lp*-LNPs^DiR^ (1 mg/mL)*, S. aureus* WH^GFP^ (1×10^7^ CFU/mL) and 293T cell^Hoechst^ (10^5^ bacteria/mL) were mixed and co-incubation for 15 min. After incubation, the samples were centrifuged (1000 rpm, 5 min). The bottom 293T cell^Hoechst^ were resuspended in PBS, and the supernatant (containing bacteria) were collected separately. Finally, both the resuspended 293T cells and supernatant bacteria were analyzed by flow cytometry.

*Quartz Crystal Microbalance with Dissipation (QCM-D) measurement:* In the QCM experiments, 200 μL of *S. aureus* (1 × 10^9^ bacteria/mL in PBS) was added to the chip (Renlux Crystal, China) and allowed to incubate for 30 minutes. The liquid was then removed using a spin coater (2000 rpm, 90s, Schwan technology, China). C-LNPs and C-*Lp*-LNPs were diluted to 0.1 mg/mL for examination using the QCM instrument. The peristaltic pump flow rate was set to 50 μL/min. Initially, PBS solution was passed through to establish a baseline and ensure the stability of the measurement environment. Following this, LNP suspensions were introduced, and after the values stabilized, PBS solution was again passed through to verify the reversibility and stability, as well as to eliminate any potential effects from non-specific adsorption or residues. Data analysis was subsequently conducted using QTools software.

To evaluate the binding capacity of *Lp*-LNPs with peptidoglycan, 200 μL of peptidoglycan solution (1 mg/mL in ultrapure water) was first added to the chip (Renlux Crystal, China) and allowed to incubate for 12 hours to form a thin film. The chip was washed with PBS three times to remove the nonadhering peptidoglycan. The subsequent experimental steps adhered to the same protocol as previously outlined. The binding capacity of *Lp*-LNPs with dextran or Glypican-3 was investigated similarly.

## *Binding of Lp-LNPs to Bacterial Biofilms*

To grow the 2-d biofilms, first, 800 μL of *S. aureus* Xen36 (2 × 10^8^ bacteria/mL in TSB) was added to a laser confocal dish (35 mm, NEST) and incubated at 37 °C for 1 hour to allow the bacteria to adhere. After the supernatant was removed, the dish was washed with PBS three times to remove the nonadhering bacteria. 1 mL TSB medium was added into the dish and cultured it for 24 h at 37 °C. The TSB medium was replaced with fresh TSB and the biofilm was grown for another 24 h. Subsequently, the supernatant was discarded, and 100 μL of SYTO^TM^ 9 dye was added, followed by incubation at 37 °C for 30 minutes. Then, 200 μL of DiR-LNPs and DiR-*Lp*-LNPs (2 mg/mL) with 10C and 14C length were added and incubated at room temperature for 2 hours. The supernatant was discarded, and the dish was washed three times with PBS before proceeding with CLSM imaging using a Nikon A-1 confocal microscope (Nikon, Japan).

## *Antibacterial Effects In Vitro*

*Measurement of minimum inhibitory concentration (MIC) and minimum bactericidal concentration (MBC):* To determine the MIC and MBC, suspensions of LNPs with different concentrations were applied to 100 μL of the bacterial suspension (2×10⁵ bacteria/mL). The plate was incubated at 37°C for 24 hours. Absorbance at 600 nm (OD_600_) of each well was measured using a microplate reader. The MIC value was defined as the lowest drug concentration, yielding an OD_600_ of less than 0.1. Subsequently, the MBC values were determined by plating aliquots of suspensions with concentrations yielding no visible growth of bacteria on agar plates after being incubated for 24 h at 37 °C, and the lowest concentration at which colony formation remained absent was taken as the MBC.

*Studying the bactericidal kinetics.* To evaluate the effects of different formulation on bacterial growth, the antibacterial process was evaluated by the standard plate counting method. *S. aureus* Xen36 or *E. coli* wu3749 (1×10^8^ bacteria/mL) suspended in 10 mM phosphate buffer (pH 6.5, ATP: 5mM, H_2_O_2_: 100 μM) was treated with: (I) PBS; (II) C-LNPs; (III) Cip; (IV) C-*Lp*-LNPs. The concentration of Cip in different formulations was 3.9 μg/mL. At 2, 4, 6, and 8 h after treatment, aliquots (10 μL) were taken, serially diluted, plated on TSB (LB for *E. coli*) plates, and incubated for 24 h at 37 °C, after which the number of CFUs formed was counted.

*CLSM* *imaging:* *S. aureus* Xen36 (1×10^8^ bacteria/mL) was treated with various formulations at a Cip concentration of 3.9 µg/mL for 4 h. Subsequently, the samples were stained with SYTO™ 9/PI fluorescent dyes and observed on a Nikon A-1 (Nikon, Japan) confocal laser scanning microscope. An argon-ion laser at 488 nm was used to excite SYTO™ 9 (green fluorescent), and a HeNe laser at 543 nm was used to excite propidium iodide (red fluorescent), collecting fluorescence at 500–535 nm (SYTO™ 9) and 583–688 nm (PI).

*TEM imaging:* To visualize the bacterial morphology, a silicon wafer (L×W×H, 5 ×5 × 0.45 mm^3^) was immersed in bacteria suspension (1×10^8^ bacteria/mL) after various treatments (at Cip concentration of 3.9 µg/mL) in a 48-well plate for 8 h to allow bacterial adhesion. The bacteria were then fixed with a 4% paraformaldehyde (Macklin, Beijing, China) for 40 minutes and then dehydrated with ethanol solutions with increasing concentrations (30%, 50%, 70%, 90%, and 100%) for 20 min successively. The silicon wafer was dried at 4 °C, sprayed with gold, and observed on a field-emission scanning electron microscope.

*Studying the bactericidal mechanism: S. aureus* Xen36 (1×10^8^ bacteria/mL) suspended in 10 mM phosphate buffer (pH 6.5, ATP: 5mM, H_2_O_2_: 100 μM) was treated with different formulations at Cip concentration of 3.9 µg/mL for 8 hours.

For β-Gal and BCA detection, the bacterial suspension was centrifuged at 15,000 rpm for 10 minutes at 4°C, and the supernatant was collected and kept on ice for further analysis. A β-Gal detection kit (Beyotime, Shanghai, China) was used for β-Gal detection using collected supernatant. A BCA detection kit (Beyotime, Shanghai, China) was used for BCA (Bicinchoninic Acid) detection using collected supernatant.

For glutathione peroxidase (GPx) detection, the bacterial suspension was subjected to ultrasonic disruption at 200 W for 3 seconds, with 10-second intervals, repeated 30 times. The mixture was then centrifuged at 15,000 rpm for 10 minutes at 4°C, and the supernatant was kept on ice for analysis using a GPx detection kit (Beyotime, Shanghai, China).

For DNase detection, the same ultrasonic disruption and centrifugation steps were employed, and the supernatant was analyzed using a DNase activity fluorescence detection kit (Beyotime, Shanghai, China).

*Intracellular ROS assay:* An aliquot of 5 mL PBS containing DCFH-DA (a reactive oxygen fluorescent probe, 20 µM) was added to an equal volume of bacterial suspension (*S. aureus* Xen36, 2 × 10^7^ bacteria/mL). After incubation for 30 min in the dark, the bacterial cells were washed with PBS twice to remove DCFH-DA that was not internalized by bacteria. The bacteria were diluted in PBS to a cell density of 10^7^ bacteria/mL. An aliquot of 50 μL of the bacterial suspension was placed in a 96-well plate, then 50 μL of PBS, C-LNPs, Cip_,_ C-*Lp*-LNPs with an equal Cip concentration of 4 μg/mL were added to the bacterial suspension, respectively. After incubation at 37 °C for 3 h, the fluorescence intensity (*Ex*=488 nm*, Em*=525 nm) was recorded on a Varioskan LUX microplate reader (ThermoFisher, USA).

*Membrane depolarization: S. aureus* Xen36 (10^7^ bacteria/mL) was mixed with DiS-C_2_(5) at a concentration of 5 μM in PBS in 96 well-plates. The resulting mixture was incubated at room temperature for 10 min, followed by 37 °C for 30 min. Then PBS, C-LNPs, Cip_,_ C-*Lp*-LNPs with an equal Cip concentration of 4 μg/mL (100 µL) were added. An excitation wavelength of 600 nm and an emission wavelength of 660 nm was used to monitor depolarization at different time intervals.

$$\text{Depolarization (\%) =}\frac{\text{FI}_{\text{treatment}}}{\text{FI}_{\text{control}}}\text{×100\%}$$

Where FI denotes fluorescence intensity, FI_treatment_ is the fluorescence intensity of bacteria after various treatments, and FI_control_ is the fluorescence intensity of DiS-C_2_(5) in PBS (5 μM).

## *Eradication of Mature Biofilms*

*CLSM imaging:* A 2-d-old biofilm was cultured with aforementioned method. The biofilms were immersed in culture medium (pH 6.5, supplemented with 5 mM ATP, 100 μM H_2_O_2_) and treated with the following formulations at 37 °C for 8 h: (I) PBS; (II) C-LNPs; (III) Cip; (IV) C-*Lp*-LNPs. The Cip concentration in different formulations was 3.9 µg/mL). After treatment, the supernatants were removed, and the biofilms were washed with PBS (500 μL × 3). Subsequently, the samples were stained with SYTO™ 9/PI fluorescent dyes for 30 min and fixed with a 4% fixative solution (Solarbio, Beijing, China), observed on a Nikon A-1 (Nikon, Japan) confocal laser scanning microscope. An argon-ion laser at 488 nm was used to excite SYTO™ 9 (green fluorescent), and a HeNe laser at 543 nm was used to excite propidium iodide (red fluorescent), collecting fluorescence at 500–535 nm (SYTO™ 9) and 583–688 nm (PI). ImageJ software (version 1.8.0) was used to reconstruct the 3D images of biofilms and analyze the fluorescence intensity and the roughness of biofilms in each confocal plane.

*CFU enumeration:* In a parallel experiment, the remaining biofilms after treatment were dispersed with PBS (1 mL), homogenized by a pipet, serially diluted, and spread on TSB agar plates. The bacterial colonies grown overnight on the agar plates were recorded and compared. The biofilms treated with acetate buffer served as the control.

*Quantifying the biomass:* In a parallel experiment, biofilms, after various treatments as mentioned above, were stained with crystal violet (0.5%, w/v) for 20 min. Crystal violet solution was removed, and biofilms were gently washed with PBS three times. 1 mL of 33% acetic acid was added to resuspend the stained biofilm for 15 min. The absorbance of each sample was recorded by a microplate reader (Spark, TECAN, Switzerland) at 595 nm.

## *Cytotoxicity Assessment*

L929 cells were used to evaluate the cytotoxicity *in vitro* by CCK-8 assay. First, 100 μL L929 cell suspension (cell concentration: 1 × 10^5^ cells/mL) was seeded into a 96-well plate. After 24-h incubation under standard culture conditions, the culture medium was replaced by a fresh medium containing C-*Lp*-LNP solutions of various concentrations (with Cip concentration ranging from 0.49 to 250 μg/mL). After 24 h, the medium was withdrawn, and the cells were washed twice with PBS. Ultimately, 100 μL CCK-8 solution was added to each sample. After another 1.5 h of incubation, the absorbance of each sample at 450 nm was measured using a microplate reader (Spark, TECAN, Switzerland). The cell viability (%) was evaluated based on the following equation:

Cell Viability (%) = [(*A*_s_ - *A*_b_) / (*A*_c_ - *A*_b_)] × 100%

where *A*_s_, *A*_b_, and *A*_c_ indicate the optical density (OD) value of the sample, blank, and control, respectively.

## *Hemolysis Assessment*

Red blood cells (RBCs) were isolated from mouse whole blood and washed three times with PBS before use. Subsequently, 0.5 mL of RBCs (5% suspended in saline) were mixed with 0.5 mL of C-*Lp*-LNPs at Cip concentrations ranging from 0.49 to 250 μg/mL and then incubated at 37°C for 1 hour. 10% Triton X-100 in ultrapure water served as the positive control, while saline was used as the negative control. The resulting mixtures were centrifuged at 4500 rpm for 15 minutes to remove RBCs. The absorbance of released hemoglobin in the supernatant was measured at 540 nm, and the hemolysis ratio was calculated using the following equation:

Hemolysis Ratio (%) = (*A*_s_ – *A*_n_) / (*A*_p_ – *A*_n_) × 100%

where *A*_s_ represents the absorbance of RBCs exposed to C-*Lp*-LNPs, *A*_n_ denotes the absorbance of RBCs exposed to saline, and *A*_p_ signifies the absorbance of RBCs exposed to 10% Triton X-100 in ultrapure water.

## *Acute Peritonitis Model*

An *S. aureus* Xen36 peritonitis infection model was established in the abdomen of female mice (6 weeks old, weighing 25-28 g). Mice were randomly divided into four groups (9 mice per group) by intraperitoneal injection of *S. aureus* Xen36 (300 μL, 2×10^8^ bacteria/mL). Two hours later, the mice were injected intraperitoneally with PBS (100 μL, Group A, control group), C-LNPs (100 μL, Group B), Cip (100 μL, Group C), or C-*Lp*-LNPs (100 μL, Group D) at equal Cip concentration of 3.9 µg/mL. Subsequently, in vivo bioluminescence imaging of the bacteria was conducted on IVIS (PerkinElmer, America) over a 0-24 hours. After 24 hours, blood was collected from the retroorbital vasculature for complete blood count analysis. ELISA kits were used to quantify the anti-inflammatory factors in the blood, along with measuring major hematology parameters. After euthanizing the mice, 1 mL of PBS was injected intraperitoneally into each mouse, followed by abdominal massage to collect the lavage fluid. Part of the peritoneal fluid was serially diluted and plated on TSB agar plates for CFU enumeration. Another portion of the peritoneal fluid was used for detecting anti-inflammatory factors. Major organs (heart, liver, spleen, lungs, kidneys) were collected, and a portion was homogenized, serially diluted, and plated on TSB agar for CFU enumeration. The remaining organs were fixed in 10% formalin, decalcified, paraffin-embedded, and subjected to H&E staining.

## *Chronic Subcutaneous Infection Model*

A bioluminescent *S. aureus* Xen36 strain (100 μL, 1×10^9^ CFU/mL) was subcutaneously injected into both hind legs of female ICR mice (6 weeks old, 25–28 g) to establish the infection model. Infected mice were randomly divided into four groups (*n* = 15 per group): PBS, C-LNPs, Cip, and C-*Lp*-LNPs. Treatments were initiated 1 day post-infection and administered every other day via localized injection at the infection site. All Cip-containing formulations were dosed at 15.6 mg/kg. Subsequently, *in vivo* bioluminescence imaging of the bacteria was conducted on IVIS (PerkinElmer, America) over a 0–7 days period. After 3 days and 7 days post-treatment, blood was collected from the mice's eyes for complete blood count analysis, and ELISA kits were used to quantify the inflammatory factors in the blood, along with measuring major blood parameters. After euthanizing the mice, the infected tissue was collected and grinded. A portion of the homogenized tissue was used for detecting inflammatory factors and the other part was serially diluted, and plated on TSB agar for CFU enumeration. The major organs (heart, liver, spleen, lungs, kidneys) were collected on days 7 and 14, and fixed in 10% formalin, decalcified, paraffin-embedded, and subjected to H&E staining.

## *Statistical Analysis*

Statistically significant differences between two specific groups were analyzed using the student's *t*-test. The statistically significant differences between three or more groups were analyzed by one-way ANOVA. *ns, p* ≥ 0.05, * *p* < 0.05, ** *p* < 0.01, *** *p* < 0.001, and **** *p* < 0.0001.

# Supplementary Figures

## Figure S1.

Synthetic route of the *Lp*-lipids used in this study.


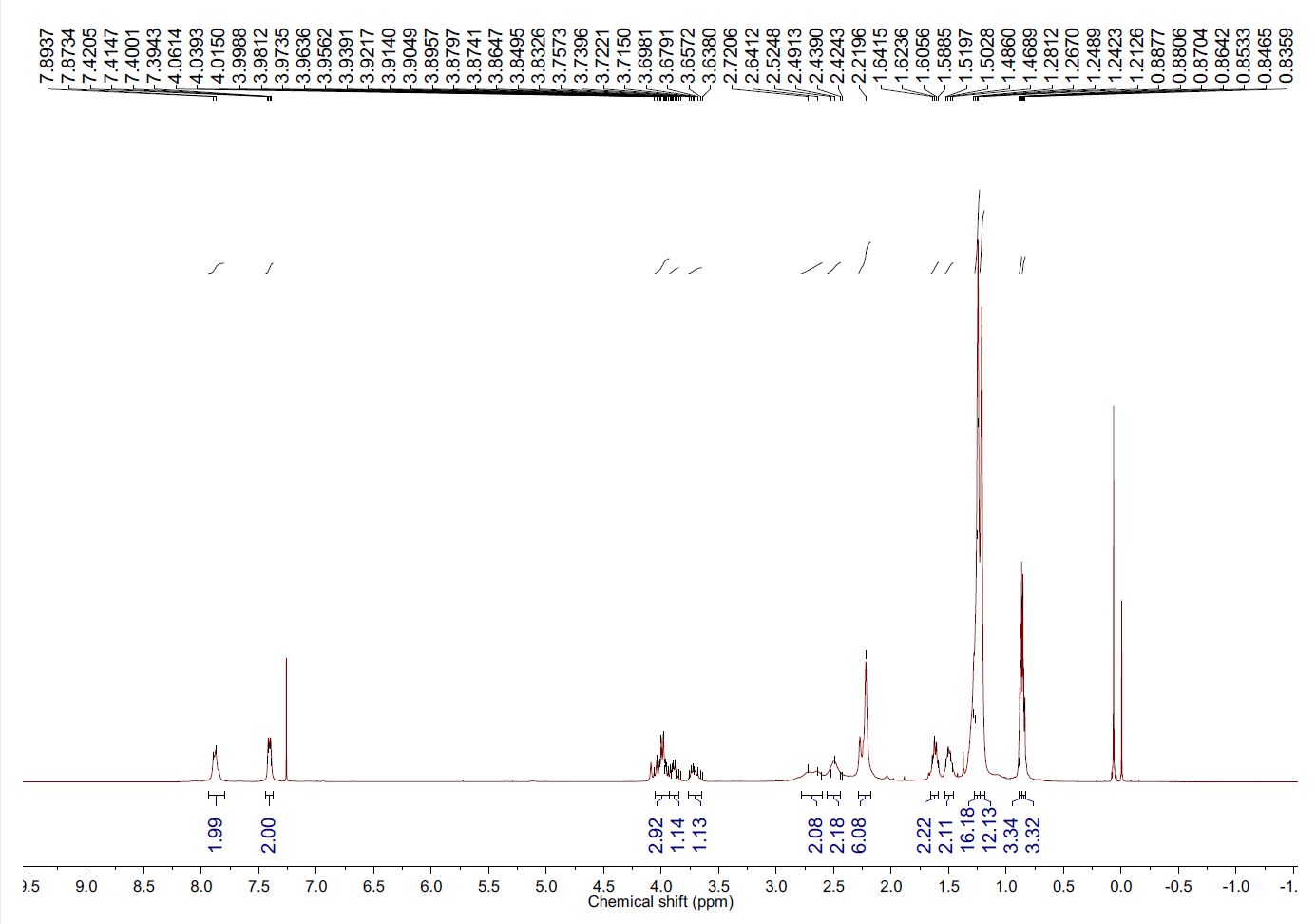


## Figure S2.

^1^H NMR spectrum of *Lp*-10C-lipid molecule in CDCl_3_ at 273K.


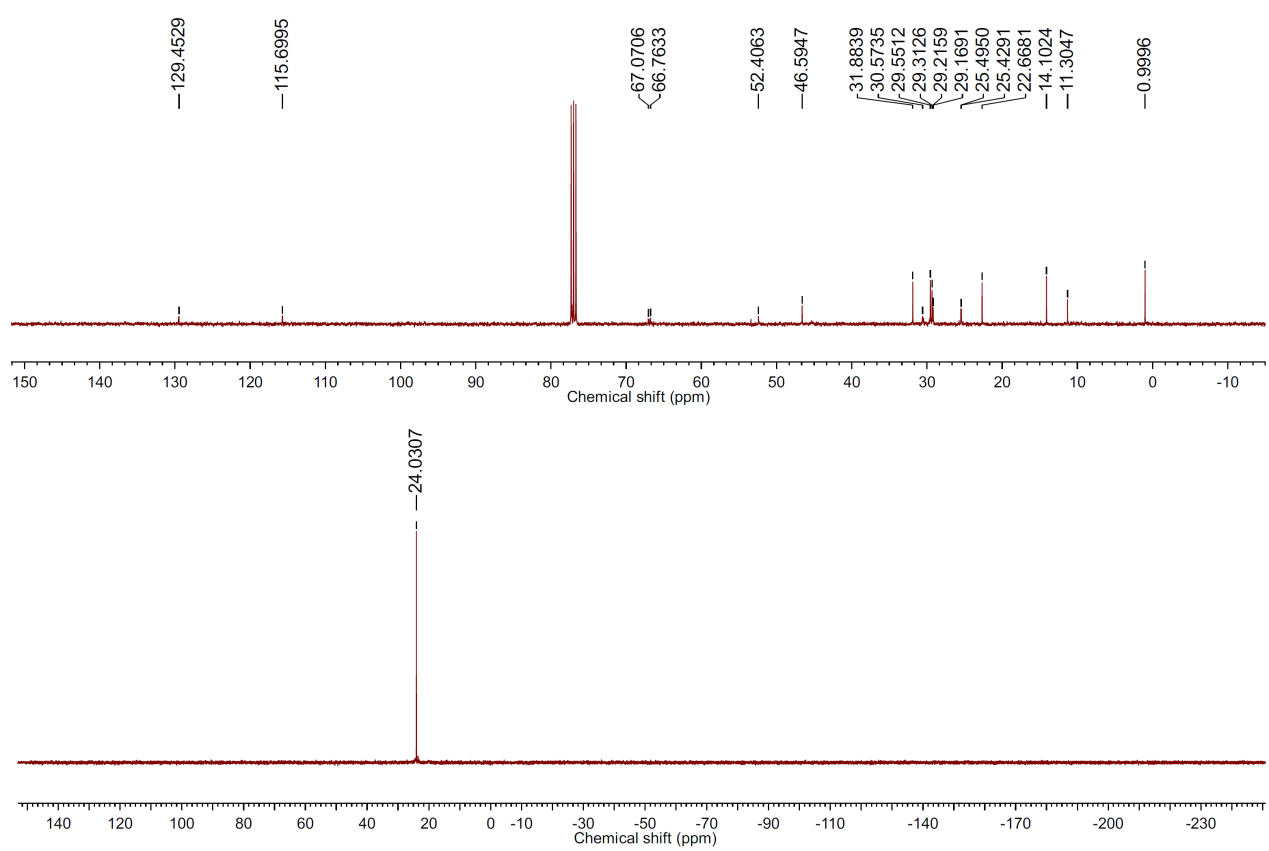


## Figure S3.

^13^C NMR and ^31^P NMR spectra of *Lp*-10C-lipid molecule in CDCl_3_ at 273K.


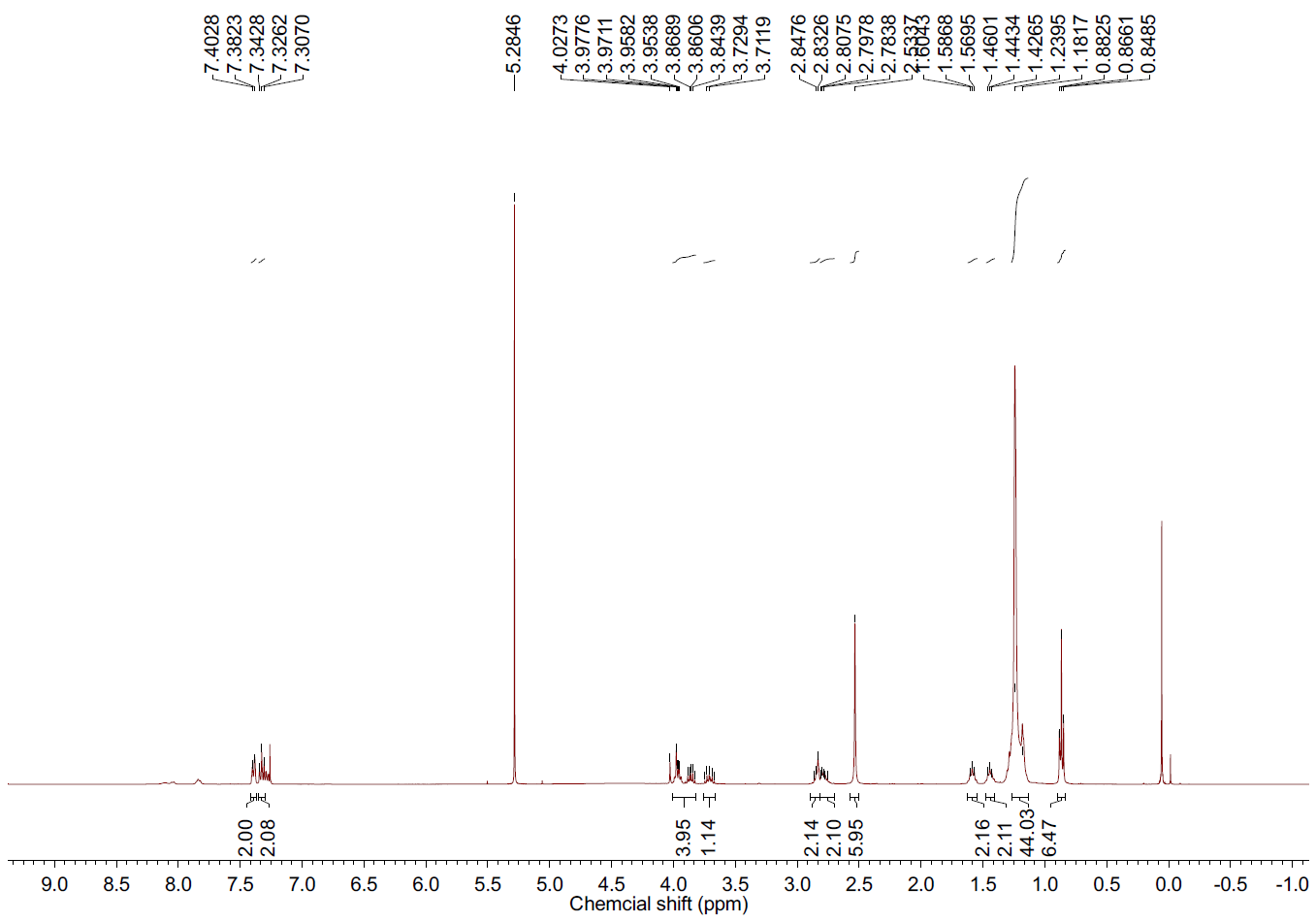


## Figure S4.

^1^H NMR spectrum of *Lp*-14C-lipid molecule in CDCl_3_ at 273K.


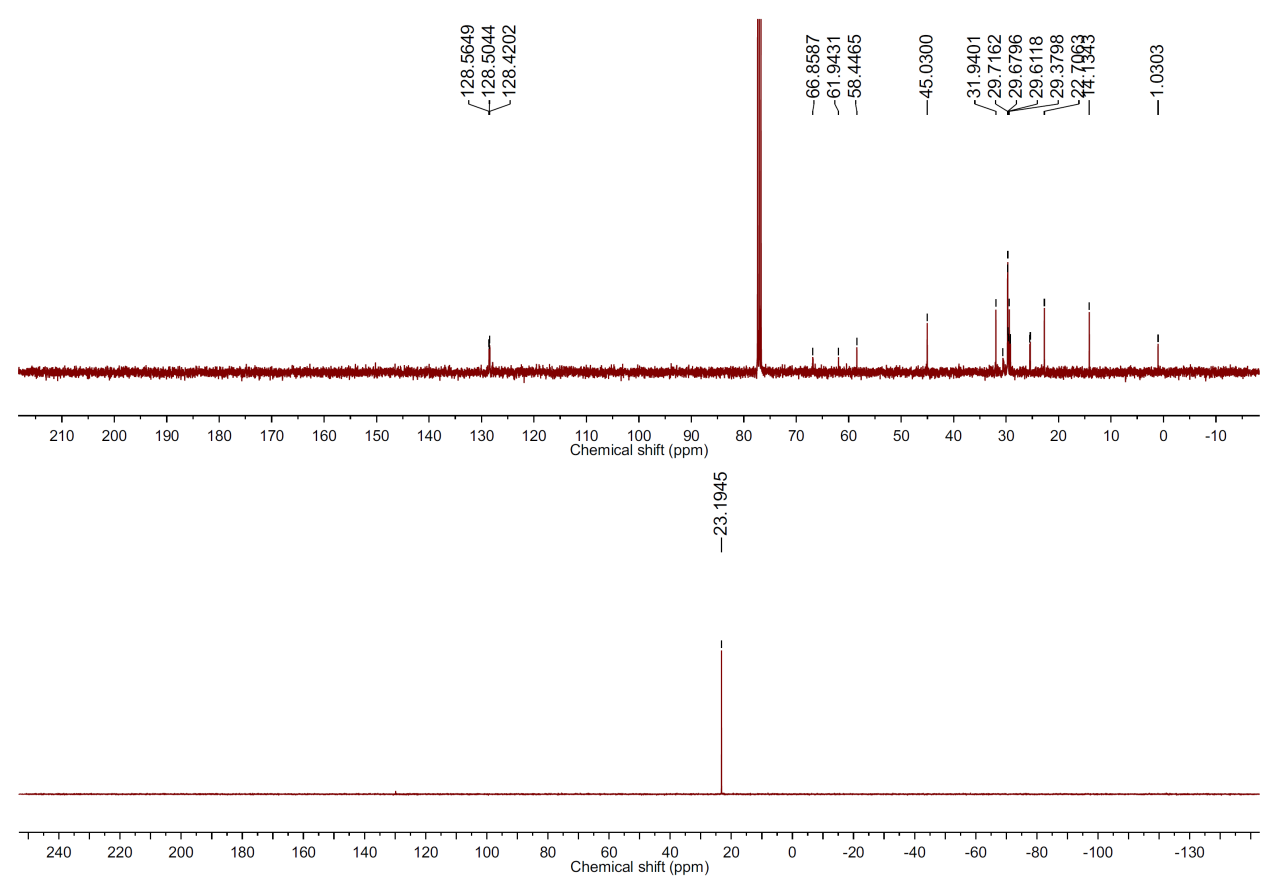


## Figure S5.

^13^C NMR and ^31^P NMR spectra of *Lp*-14C-lipid molecule in CDCl_3_ at 273K.


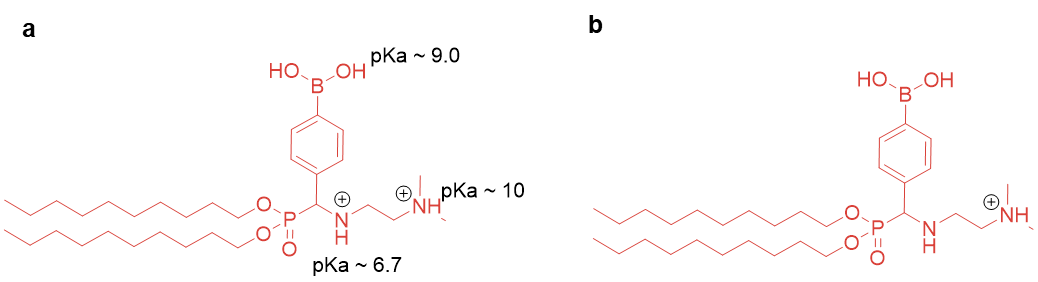


## Figure S6.

(**a**) The estimated pKa value of the key domains in the lipid used in this study. (**b**) Chemical structure showing the protonation status of the lipid at pH 7.4.

**
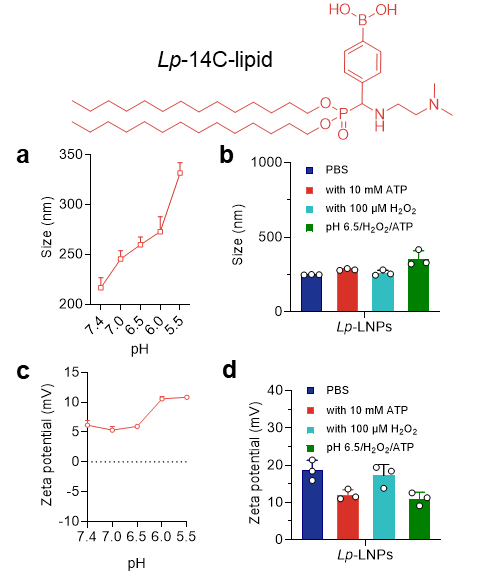
**

## Figure S7.

(**a**) Size changes of *Lp*-LNPs composed of *Lp*-14C lipids after exposure to pH levels ranging from 7.4 to 5.5. (**b**) Size changes of *Lp*-LNPs composed of *Lp*-14C lipids in the absence or presence of 5 mM ATP, 100 µM H₂O₂, and pH 6.5/H₂O₂/ATP for 2 hours, emulating the acidic/oxidative/ATP microenvironment present in bacterial infections. (**c**) Zeta potentials of *Lp*-LNPs composed of *Lp*-14C lipids after exposure to pH levels ranging from 7.4 to 5.5. (**d**) Zeta potential changes of *Lp*-LNPs in the absence or presence of 5 mM ATP, 100 µM H₂O₂, and pH 6.5/H₂O₂/ATP for 2 hours, emulating the acidic/oxidative/ATP microenvironment present in bacterial infections. Size and zeta potentials were measured using the dynamic light scattering method, with the *Lp*-LNP concentration set at 100 µg/mL during the measurements. Data are presented as mean ± standard deviations over three replicates.


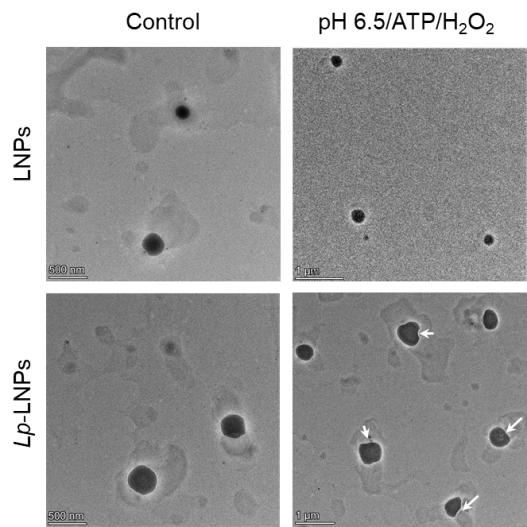


## Figure S8.

Representative TEM images (with multiple nanoparticles) of the LNPs and *Lp*-LNPs before and after exposure to pH 6.5/H₂O₂/ATP for 2 hours, with white arrows indicating the defects of *Lp*-LNPs observed in the nanoparticles following the stimulation.


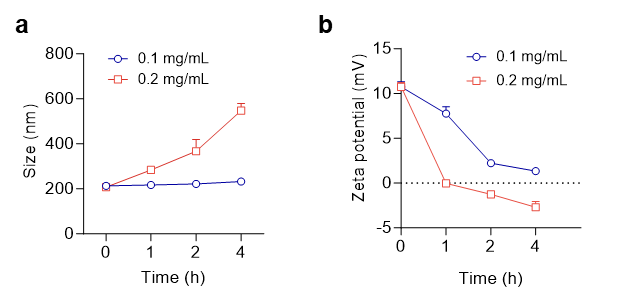


## Figure S9.

Size and Zeta potential variations of *Lp*-LNPs after exposure to glucose at different concentrations (0.1 and 0.2 mg/mL) for different time intervals (*n* = 3 independent samples).

## Figure S10.

Size variations of *Lp*-LNPs during the 5-day observation period (*n* = 3 independent samples).

## Figure S11.

The evolution of membrane thickness during the 100-ns simulation of the pure Egg PC, Egg PC+*Lp*-lipid, and Egg PC+*Lp*-lipid+ATP.

## Figure S12.

Interaction energy between Egg PC/Egg PC or Egg PC/*Lp*-lipid molecules during the 100 ns co-assembly process.


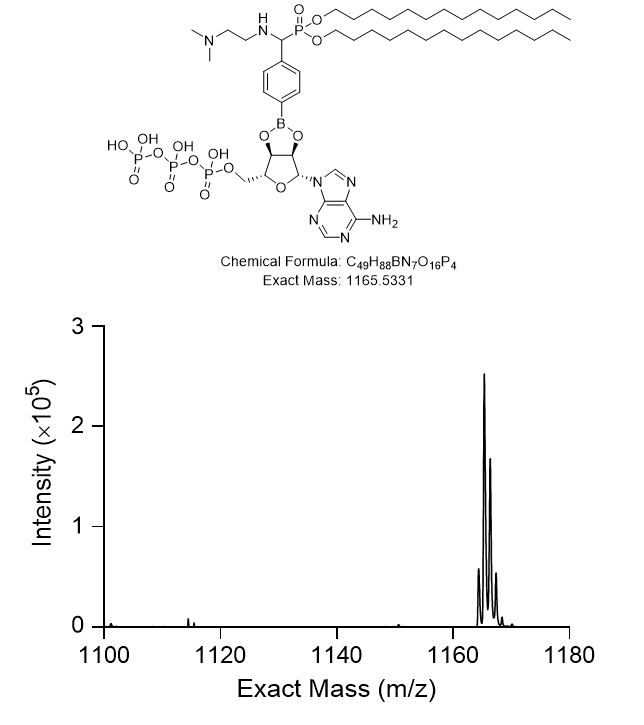


## Figure S13.

Matrix-assisted laser desorption/Ionization time-of-flight (MALDI-TOF) mass spectrometry of *Lp*-14C-lipids after exposure to ATP for 2 hours, illustrating the formation of a boronate structure between *pba* in *Lp*-14C-lipids and the cis-diol in ATP.

## Figure S14.

The proposed mechanism of *Lp*-lipid in the presence of H_2_O_2_.


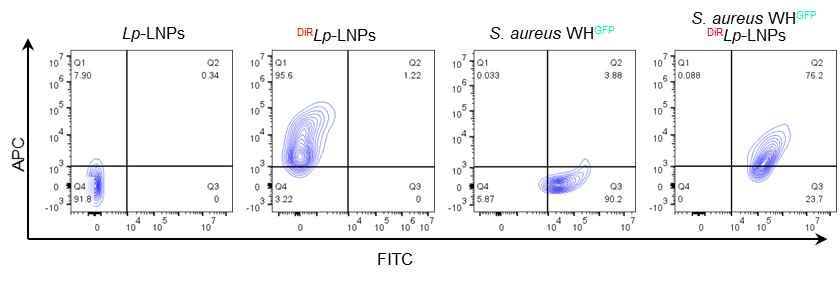


## Figure S15.

Flow cytometry analysis demonstrating the interactions of *S. aureus* WH^GFP^ with DiR-loaded *Lp*-LNPs.


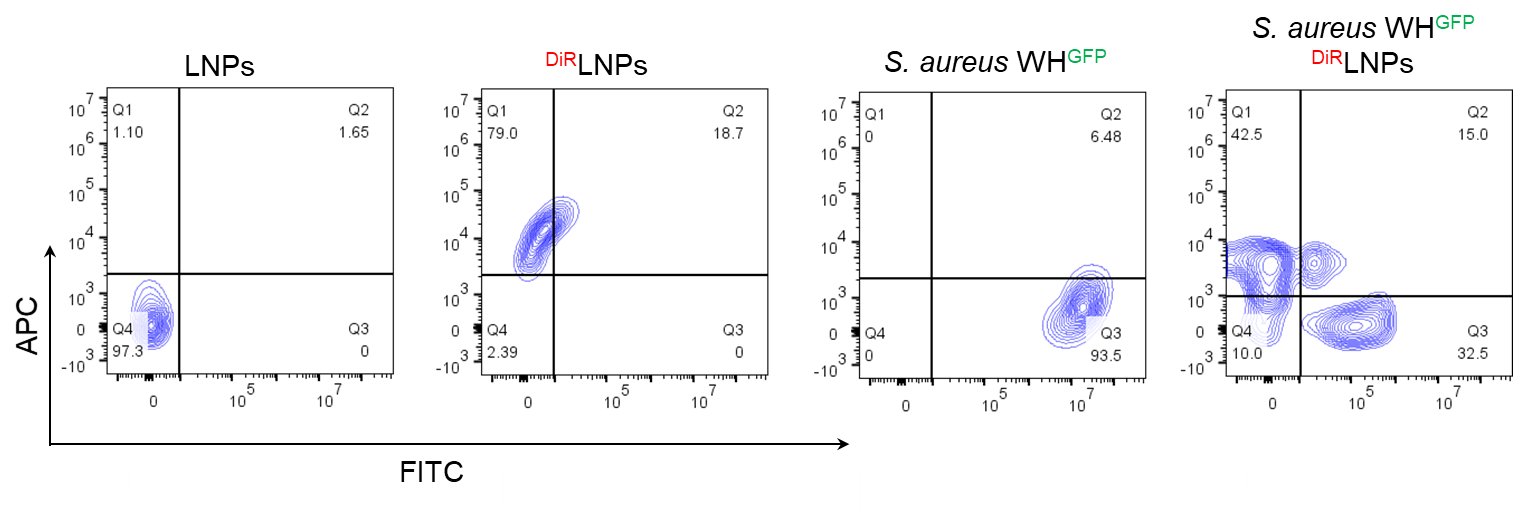


## Figure S16.

Flow cytometry analysis demonstrating the interactions of *S. aureus* WH^GFP^ with DiR-loaded LNPs.


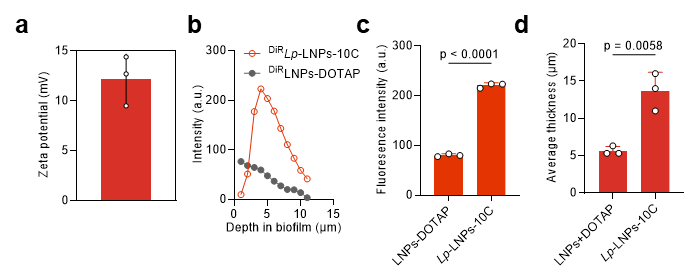


## Figure S17.

(**a**) Zeta potential of the LNPs composed of DOTAP as the key positive component. (**b**) Quantified fluorescence intensity from the CLSM planes as a function of biofilm depth after exposure to DiR-loaded LNPs-DOTAP and *Lp*-LNPs. (**c**) Total fluorescence intensity within the *S. aureus* biofilms following a 2-hour exposure to DiR-loaded LNPs-DOTAP and *Lp*-LNPs. (**d**) Average thickness demonstrating the penetration of DiR-loaded LNPs and Lp-LNPs into S. aureus biofilms.


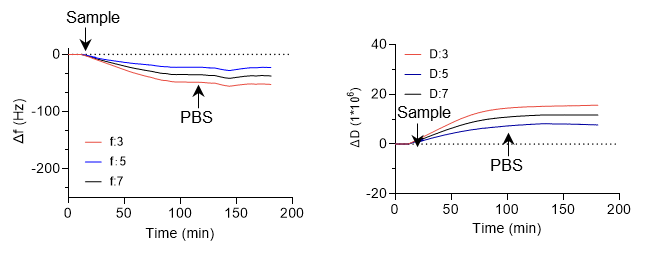


## Figure S18.

QCM-D analysis of Δf and ΔD observed for the third, fifth, and seventh overtones resulting from the adsorption of LNPs and *Lp*-LNPs on GPC3-coated sensors at a flow rate of 0.15 mL/min.


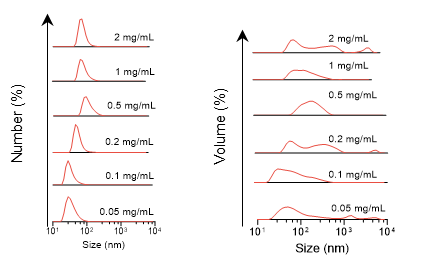


## Figure S19.

Size distribution of C-*Lp*-LNPs prepared with varying Cip loading concentrations, as measured by DLS and analyzed based on number and volume percentages, respectively.

## Figure S20.

Zeta potentials of the C-*Lp*-LNPs prepared from different Cip loading concentrations, assessed using the DLS technique


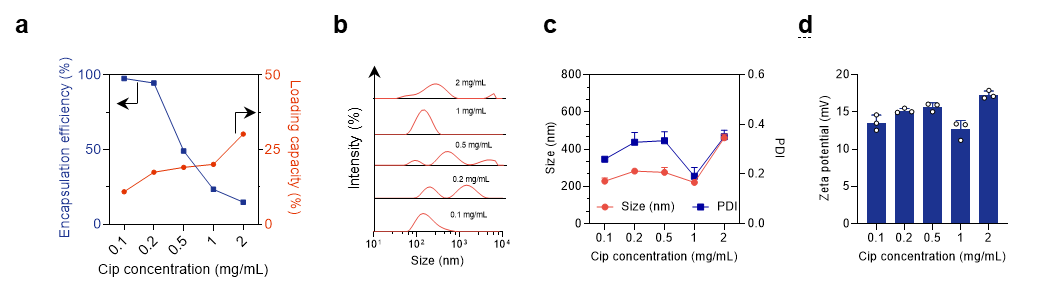


## Figure S21.

(**a**) Encapsulation efficiency and loading capacity of cip in *Lp*-LNPs composed of 14C lipid with varying ciprofloxacin feeding concentrations. (**b**) Size distribution of the C-*Lp*-LNPs composed of 14C lipid prepared from different ciprofloxacin feeding concentrations, as measured using the DLS method. (**c**) Size and polydispersity index (PDI) of the C-*Lp*-LNPs composed of 14C lipid prepared from various ciprofloxacin feeding concentrations, determined via the DLS method. (**d**) Zeta potentials of the C-*Lp*-LNPs composed of 14C lipid prepared from different ciprofloxacin feeding concentrations, assessed using the DLS technique. Data are presented as mean ± standard deviations across three replicates.


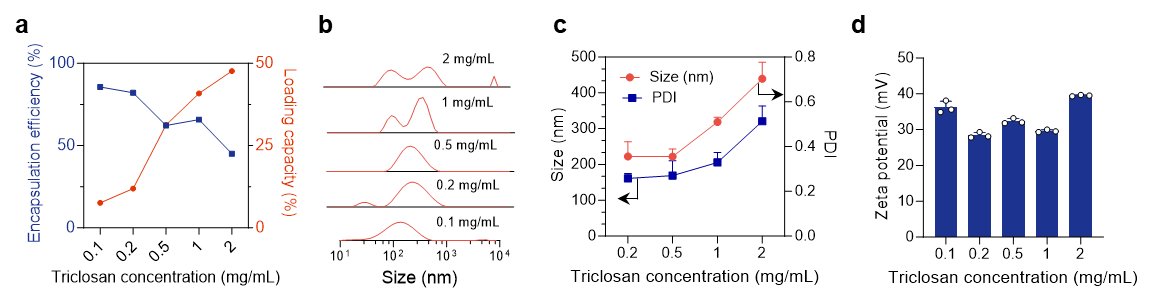


## Figure S22.

(**a**) Encapsulation efficiency and loading capacity of triclosan in *Lp*-LNPs with varying triclosan feeding concentrations. (**b**) Size distribution of the triclosan-loaded -*Lp*-LNPs prepared from different triclosan loading concentrations, as measured using the DLS method. (**c**) Size and polydispersity index (PDI) of the triclosan-loaded -*Lp*-LNPs prepared from various triclosan loading concentrations, determined via the DLS method. (**d**) Zeta potentials of the triclosan-loaded-*Lp*-LNPs prepared from different triclosan loading concentrations, assessed using the DLS technique. Data are presented as mean ± standard deviations across three replicates.


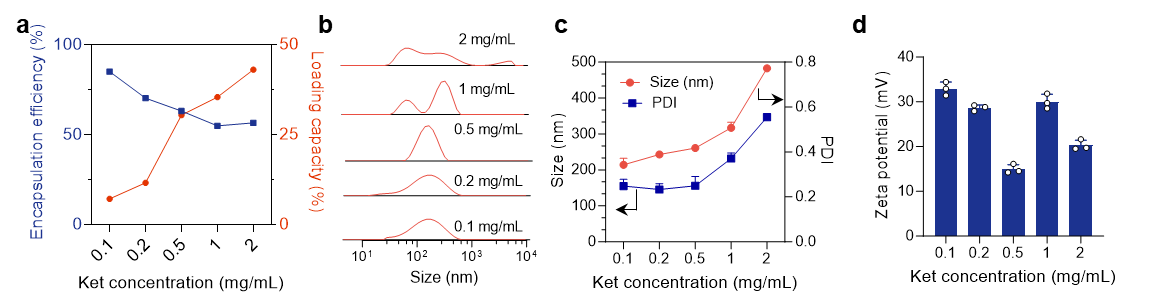


## Figure S23.

(**a**) Encapsulation efficiency and loading capacity of Ketoconazole (ket) in *Lp*-LNPs with varying ket feeding concentrations. (**b**) Size distribution of the ket-loaded-*Lp*-LNPs prepared from different ket loading concentrations, as measured using the DLS method. (**c**) Size and polydispersity index (PDI) of the ket-loaded-*Lp*-LNPs prepared from various ket loading concentrations, determined via the DLS method. (**d**) Zeta potentials of the ket-loaded-*Lp*-LNPs prepared from different triclosan loading concentrations, assessed using the DLS technique. Data are presented as mean ± standard deviations across three replicates.


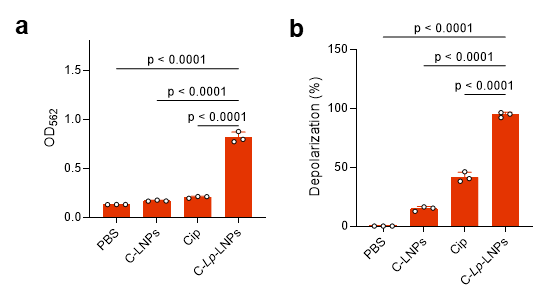


## Figure S24.

(**a**) Fluorescent intensity of intracellular ROS in *S. aureus* Xen36 after various treatments. (**b**) Membrane depolarization rate of *S. aureus* Xen36 after various treatments.


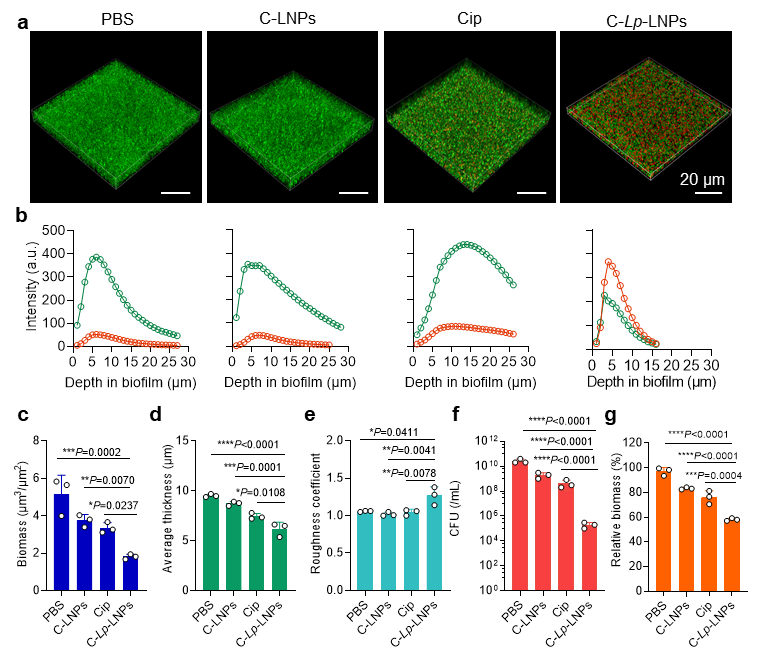


## Figure S25.

(**a**) Representative 3D CLSM images of *S. aureus* Xen36 biofilms after exposure to various treatments. (**b**) Quantified red and green fluorescence intensity in each CLSM plane as a function of depth in biofilm. (**c-e**) Biomass, average thickness, and roughness calculated via COMSTAT 2.1 Image J plugin. (**f**) CFU enumeration of *S. aureus* Xen36 biofilms after various treatments. (**g**) Relative biovolume of *S. aureus* Xen36 biofilms after various treatments and staining with crystal violet. The Cip concentration equals 3.9 μg/mL. Data are mean ± s.d of *n* = 3 biologically independent samples. Statistical significance was analyzed by one-way ANOVA with Tukey’s multiple comparisons test.

**Figure S26.**

Relative viability of L929 cells following exposure to C-*Lp*-LNPs at different concentrations, with the viability of L929 cells treated with PBS set as 100% (*n* = 3 independent samples).


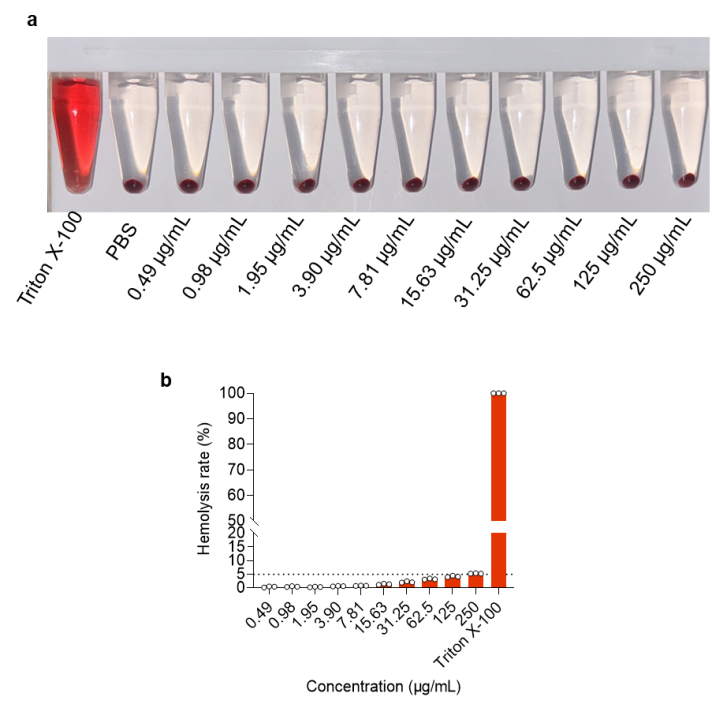


**Figure S27.**

Images and quantified data showing hemolysis rates of C-*Lp*-LNPs against red blood cells at various concentrations, with the hemolysis rate of Triton X-100 set as 100% (*n* = 3 independent samples).


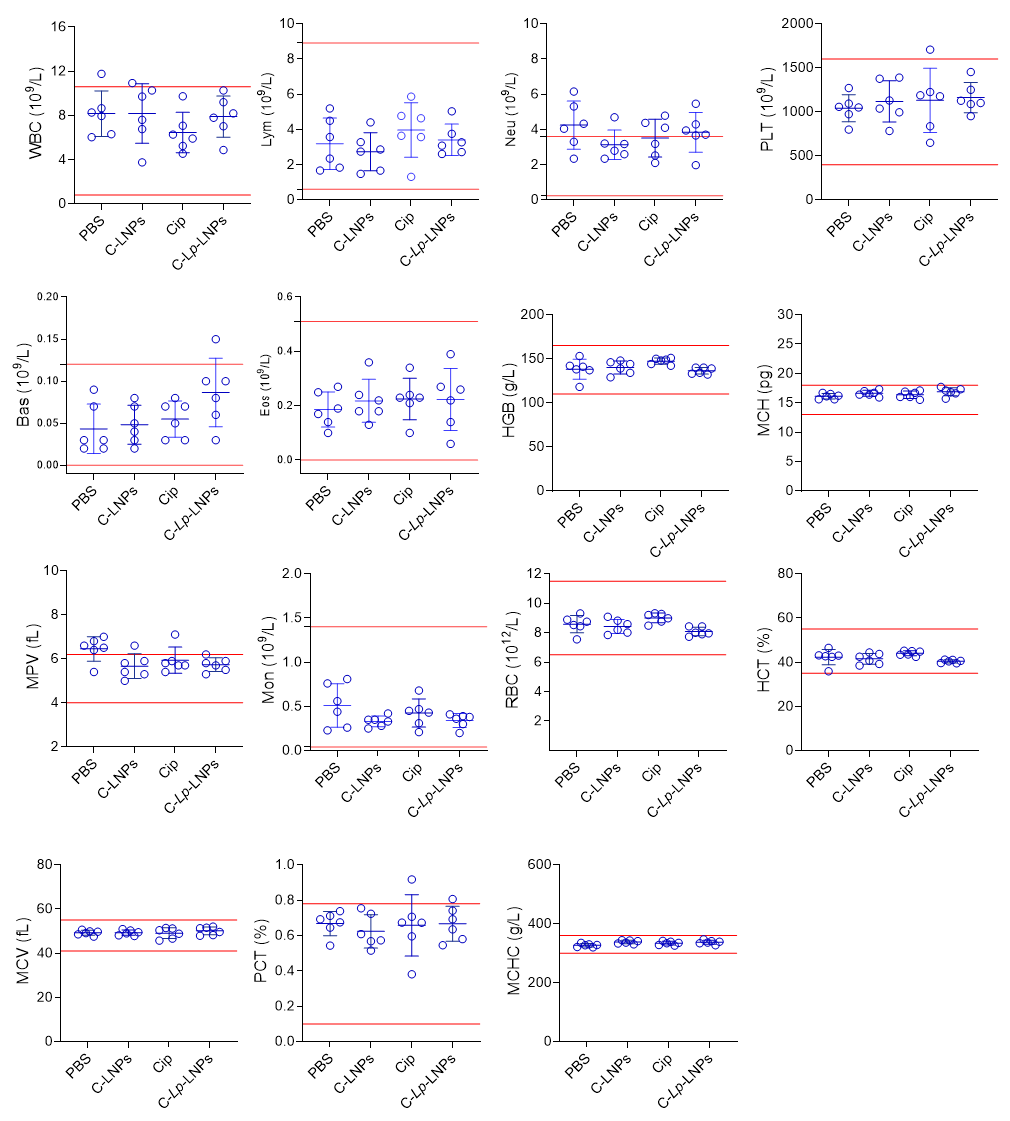


**Figure S28.**

Major hematology parameters after various treatments in the murine peritoneal infection model. Blood samples were taken at 24 hours after initiating treatment, with WBC representing white blood cells, Lym representing lymphocytes, Nue representing neutrophils, PLT representing platelets, Bas representing basophils, Eos representing eosinophils, HGB representing hemoglobin, MCH representing mean corpuscular hemoglobin, MPV representing mean platelet volume, Mon representing monocytes, RBC representing red blood cells, HCT representing hematocrit, MCV representing mean corpuscular volume, PCT representing platelet count, MCHC representing mean corpuscular hemoglobin concentration. The red lines represent the reference ranges and Error bars denote standard deviation (SD) over five mice in each group.


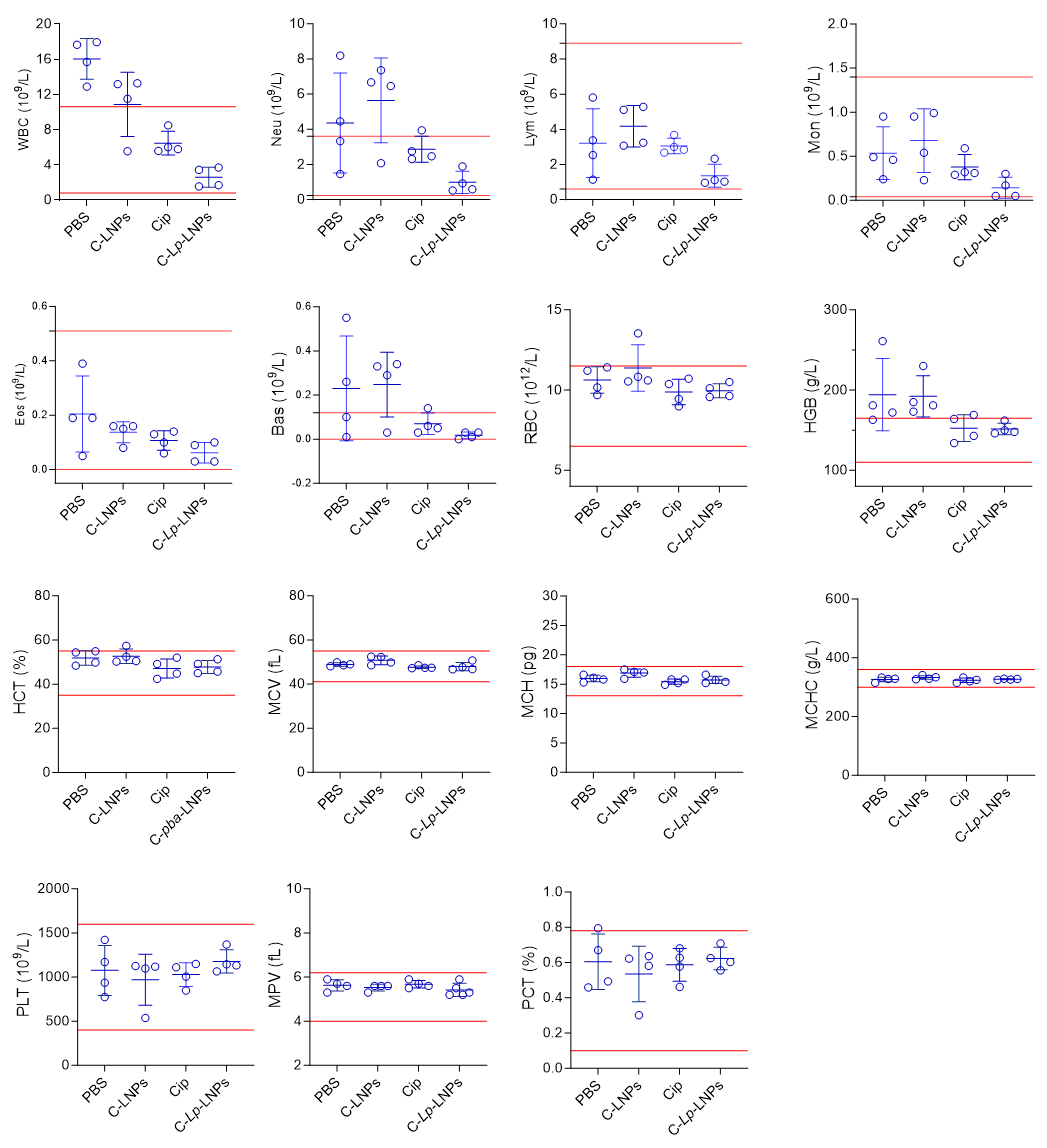


**Figure S29.**

Major hematology parameters on day 3 after various treatments in the subcutaneous infection model. Blood samples were taken at 24 hours after initiating treatment, with WBC representing white blood cells, Lym representing lymphocytes, Nue representing neutrophils, PLT representing platelets, Bas representing basophils, Eos representing eosinophils, HGB representing hemoglobin, MCH representing mean corpuscular hemoglobin, MPV representing mean platelet volume, Mon representing monocytes, RBC representing red blood cells, HCT representing hematocrit, MCV representing mean corpuscular volume, PCT representing platelet count, MCHC representing mean corpuscular hemoglobin concentration. The red lines represent the reference ranges and Error bars denote standard deviation (SD) over five mice in each group.


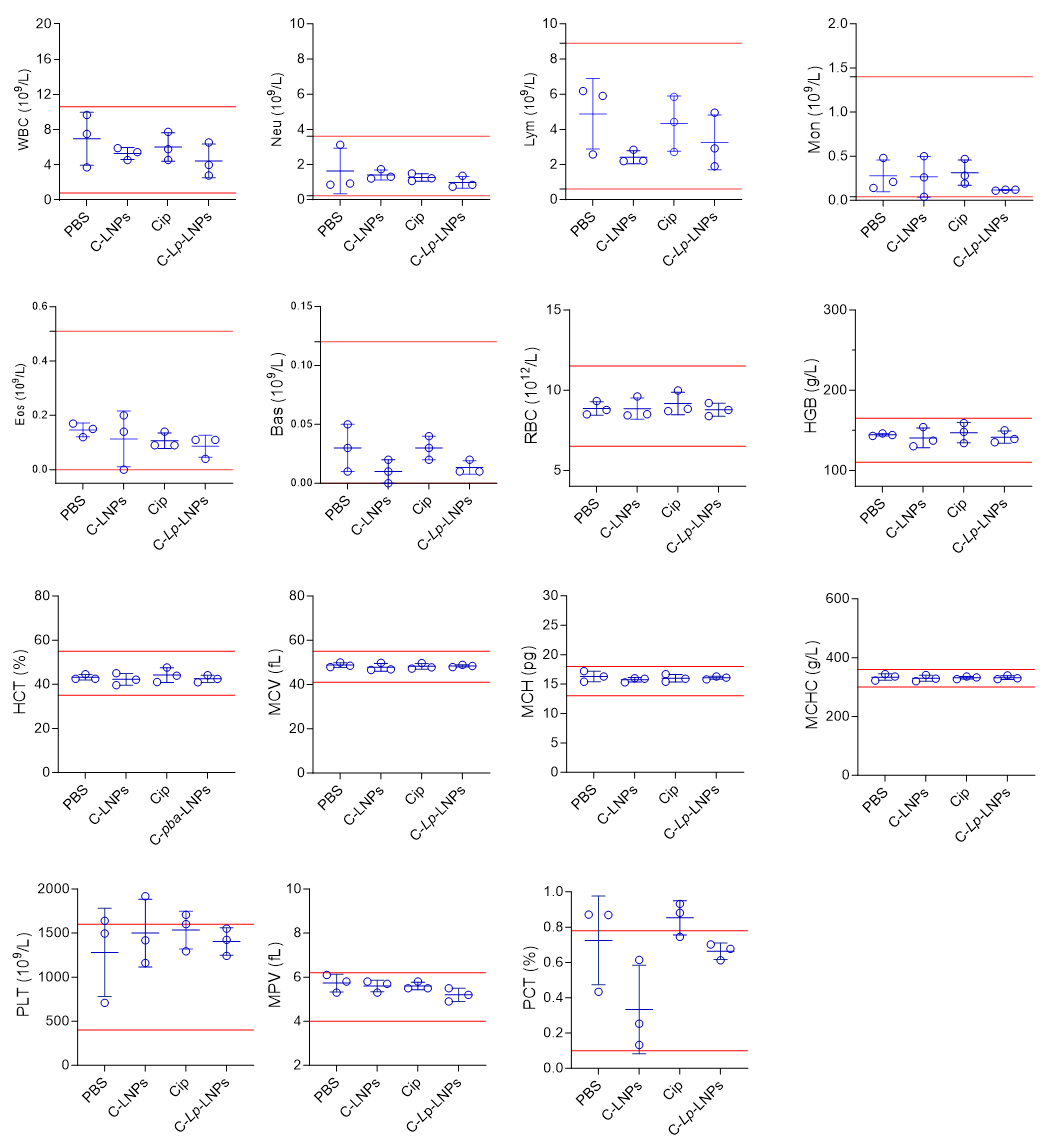


**Figure S30.**

Major hematology parameters on day 7 after various treatments in the subcutaneous infection model. Blood samples were taken at 24 hours after initiating treatment, with WBC representing white blood cells, Lym representing lymphocytes, Nue representing neutrophils, PLT representing platelets, Bas representing basophils, Eos representing eosinophils, HGB representing hemoglobin, MCH representing mean corpuscular hemoglobin, MPV representing mean platelet volume, Mon representing monocytes, RBC representing red blood cells, HCT representing hematocrit, MCV representing mean corpuscular volume, PCT representing platelet count, MCHC representing mean corpuscular hemoglobin concentration. The red lines represent the reference ranges and Error bars denote standard deviation (SD) over five mice in each group.


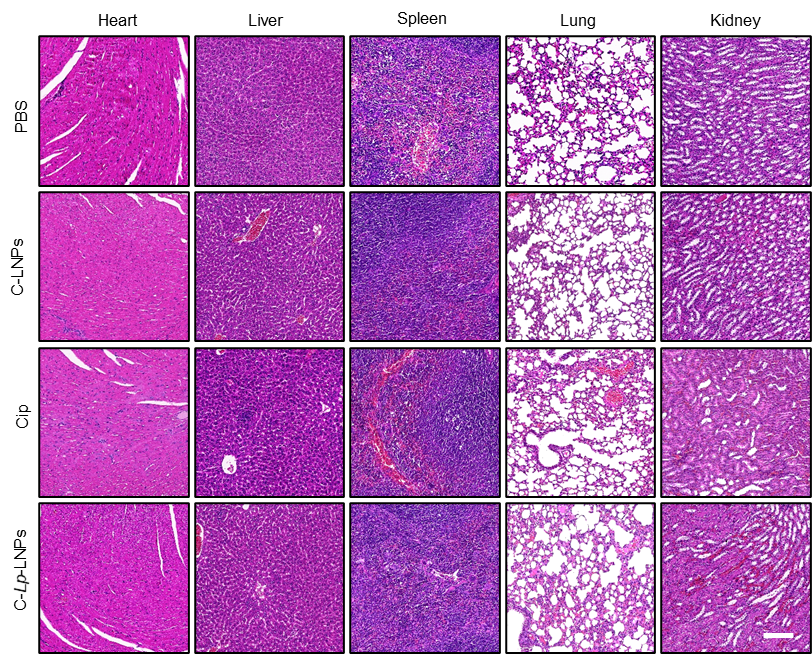


**Figure S31.**

H&E staining of major organ sections harvested from mice after various treatments in the murine peritoneal infection model at 24 hours after initiating treatments. Bar represents 200 μm.


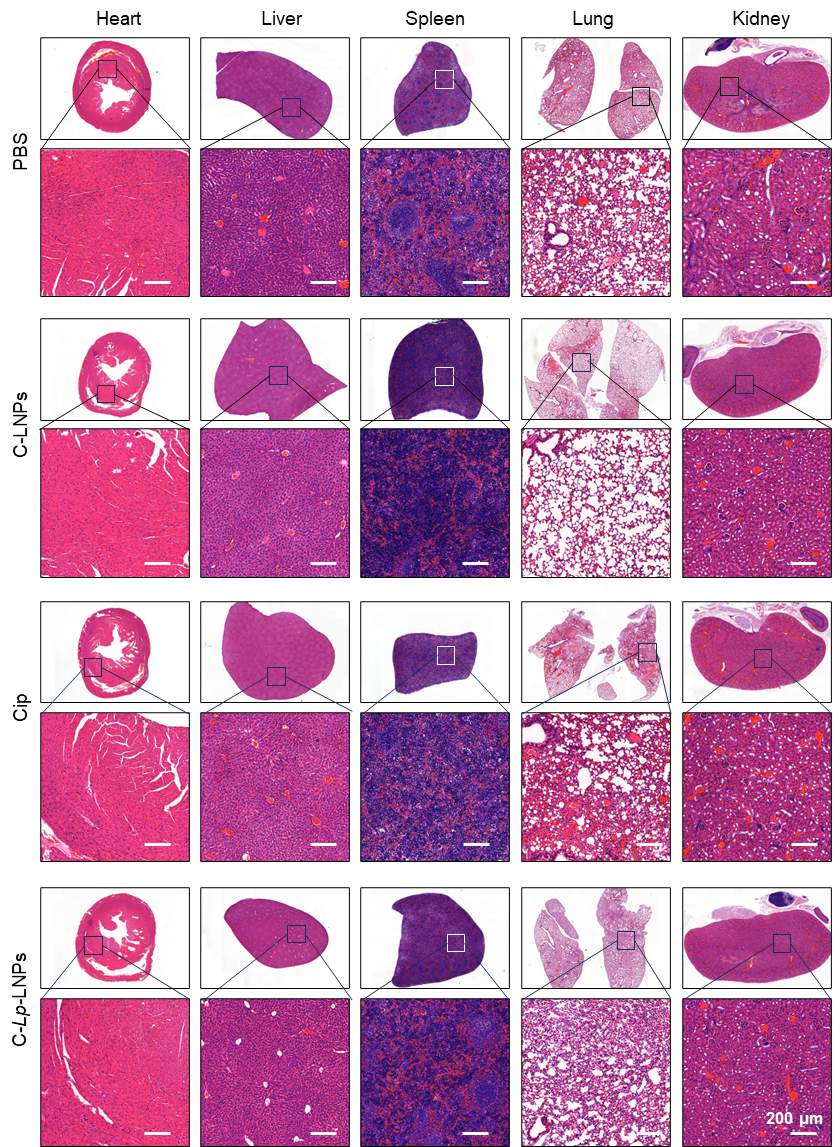


**Figure S32.**

H&E staining of major organ sections harvested from mice on day 7 after various treatments. Bar represents 200 μm.


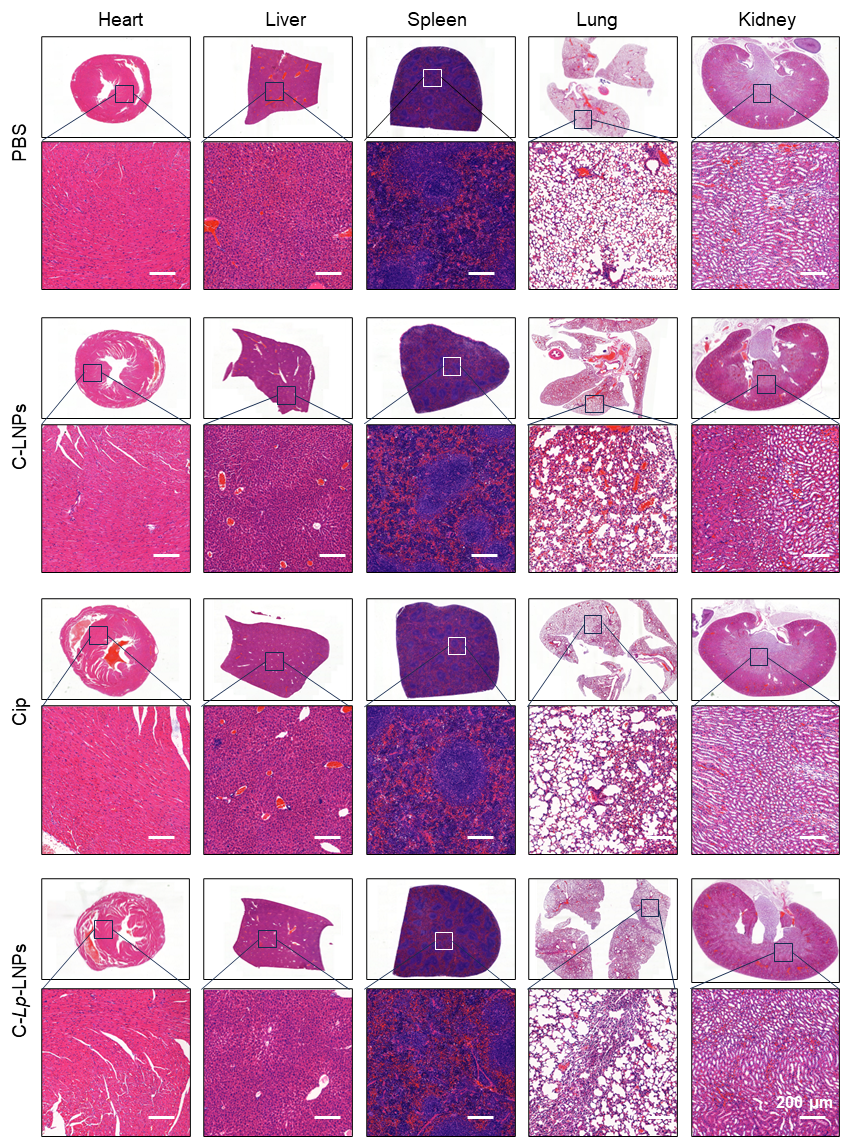


**Figure S33.**

H&E staining of major organ sections harvested from mice on day 14 after various treatments. Bar represents 200 μm.

**Table S1**

**MIC and MBC values of various formulations against *E. coli* wu3749 and *S. aureus* Xen36.**

|  | *E. coli* wu3749 | | *S. aureus* Xen36 | |
| --- | --- | --- | --- | --- |
|  | MIC (μg/mL) | MBC (μg/mL) | MIC (μg/mL) | MBC (μg/mL) |
| Cip | 1.6 | 3.12 | 1.60 | 3.12 |
| PC | >250 | >250 | > 250 | > 250 |
| C-LNPs | 62.5 | 125 | 250 | > 250 |
| *Lp*-lipids | >250 | >250 | > 250 | > 250 |
| C*-Lp*-LNPs | 0.98 | 1.95 | 0.98 | 1.95 |
